# *Salmonella* escapes adaptive immune response via SIRT2 mediated modulation of innate immune response in dendritic cells

**DOI:** 10.1371/journal.ppat.1007437

**Published:** 2018-11-19

**Authors:** Mayuri Gogoi, Kasturi Chandra, Mohsen Sarikhani, Ramya Ramani, Nagalingam Ravi Sundaresan, Dipshikha Chakravortty

**Affiliations:** 1 Department of Microbiology and Cell Biology, Indian Institute of Science, Bangalore, India; 2 Division of Biological Sciences, Indian Institute of Science, Bangalore, India; 3 Centre for Biosystems Science and Engineering, Indian Institute of Science, Bangalore, India; Duke University School of Medicine, UNITED STATES

## Abstract

*Salmonella* being a successful pathogen, employs a plethora of immune evasion mechanisms. This contributes to pathogenesis, persistence and also limits the efficacy of available treatment. All these contributing factors call upon for new drug targets against *Salmonella*. For the first time, we have demonstrated that *Salmonella* upregulates sirtuin 2 (SIRT2), an NAD^+^ dependent deacetylase in dendritic cells (DC). SIRT2 upregulation results in translocation of NFκB p65 to the nucleus. This further upregulates NOS2 transcription and nitric oxide (NO) production. NO subsequently shows antibacterial activity and suppresses T cell proliferation. NOS2 mediated effect of SIRT2 is further validated by the absence of effect of SIRT2 inhibition in NOS2^-/-^ mice. Inhibition of SIRT2 increases intracellular survival of the pathogen and enhances antigen presentation *in vitro*. However, *in vivo* SIRT2 inhibition shows lower bacterial organ burden and reduced tissue damage. SIRT2 knockout mice also demonstrate reduced bacterial organ burden compared to wild-type mice. Collectively, our results prove the role of SIRT2 in *Salmonella* pathogenesis and the mechanism of action. This can aid in designing of host-targeted therapeutics directed towards inhibition of SIRT2.

## Author summary

*Salmonella enterica* is the cause of infectious diseases which ranges from self-limiting diarrhoea to fatal systemic illness like typhoid. During its pathogenesis, *Salmonella* survives inside dendritic cells (DCs) by suppressing antigen presentation, thereby successfully evading host response. Although, various previous studies have focused on the role of host epigenetic modification during *Salmonell*a pathogenesis, till date, there has been no study on the role of SIRT2 in *Salmonella* infection. Here, we show that *Salmonella* upregulates SIRT2 expression in DCs, which in turn upregulates nitric oxide production by enhancing nuclear translocation of NFκB. Being a suppressor of T cell proliferation as well as an antimicrobial agent, nitric oxide regulation can affect *Salmonella* infection in both positive and negative ways, respectively. This study shows the trade-off made by *Salmonella* where, *in vitro* infection mediated upregulation of SIRT2 enhances antimicrobial response, but simultaneous higher intracellular NO inhibits T cell response leading to impaired antigen presentation and successful pathogenesis. Since inhibition of SIRT2 gives a fitness advantage to the infected host leading to better clearance of the pathogen, our findings may have further implications in the development of novel therapeutics.

## Introduction

Sirtuins are a family of proteins originally discovered in yeast as a homolog to silent information regulator 2 gene (Sir2). Pioneering studies on Sir2 in *Saccharomyces cerevisiae* demonstrate its deacetylase function which is essential for silencing transcription at silent mating loci, telomeres and recombination in rDNA [1]. Mammalian homologs of Sir2 belong to HDAC-III family and are of seven types (SIRT1-7). All SIRTs share a conserved NAD^+^ binding domain, a catalytic domain and a variable C- terminal domain but shows differential subcellular localization. SIRT1 shows nuclear and cytoplasmic localization, SIRT2 is predominantly present in the cytoplasm but can translocate to the nucleus upon external triggers. SIRT3, SIRT4 and SIRT5 show mitochondrial localization. SIRT6 is a chromatin-associated deacetylase and SIRT7 is present in the nucleolus. SIRTs can deacetylate both histone and non-histone substrates [2,3]. A large body of literature has suggested the role of SIRTs in aging and age-related pathologies [3].

SIRT2 is a cytosolic protein but it can translocate to the nucleus [4]. This makes it possible for SIRT2 to deacetylate both cytosolic substrates like microtubule [5] and nuclear substrates like histones [6]. SIRT2 has been implicated in various physiological processes like cell cycle, neurodegenerative diseases, oxidative stress, genome stability, cancer, arthritis, inflammation and autophagy [7]. Although the involvement of SIRT2 in oxidative stress [8,9], inflammation, microglial activation [10], macrophage polarization [11] and during sepsis [12] have been studied, the role of SIRT2 in bacterial pathogenesis yet remains largely elusive. Till date, there have been only two studies which have looked into the same. In the case of intracellular bacteria *Listeria monocytogenes*, the infection induces SIRT2 translocation to the nucleus which results in deacetylation of lysine 18 residue of histone 3. This further leads to a change in chromatin organization and hence reprogramming of host transcriptional landscape. Deletion of SIRT2 *in vivo* results in lower *Listeria* burden [13], thus, asserting its role in *Listeria* pathogenesis. Another study in *Mycobacterium tuberculosis* suggests that the deletion of SIRT2 in myeloid lineage has a transient effect on mycobacterial infection. However, it does not show any long-term effect on mycobacterial pathogenesis [14]. *Salmonella enterica* causes a vast array of diseases which ranges from self-limiting diarrhoea to systemic diseases like typhoid. *Salmonella* follows a faeco-oral route of transmission.

Recent reports have estimated 21 million typhoid cases [15] and 93 million [16] nontyphoidal *Salmonella* diseases (NTS) cases every year. *Salmonella* is an intracellular pathogen which infects various host cells and resides in a modified vacuole called *Salmonella-* containing vacuole. *Salmonella* Typhi causes systemic infection in humans, whereas *Salmonella* Typhimurium causes systemic disease in mice. A mouse model of *Salmonella* serves as an animal model to study *Salmonella* pathogenesis. During infection, *Salmonella* infects various host cells such as epithelial cells, macrophages, dendritic cells (DCs), polymorphonuclear cells. Among all these cells, dendritic cells play a crucial role in *Salmonella* pathogenesis. It is pivotal for bacterial entry as DCs present in the Peyer’s patch can directly sample luminal bacteria [17]. DCs are important for systemic dissemination of the bacteria as well. Once the bacterium crosses the intestinal epithelial layer, it enters various immune cells like macrophage, DCs, neutrophils etc. These cells can further disseminate the bacteria to other organs. DCs are also integral to host immune response. They are a member of both innate and adaptive immune responses. The reactive oxygen species (ROS) and reactive nitrogen species (RNS) play an important role in the clearance of *Salmonella* [18].

*Salmonella* has been reported to suppress DC-mediated antigen presentation, making it essential for mounting an appropriate immune response [19]. SIRT2 has already been reported to play an essential role in LPS mediated inflammatory response in macrophages [20]. It has also been implicated to play a role in oxidative [8] and nitrosative stress [12].

In this study, we have evaluated the effect of SIRT2 in *Salmonella* pathogenesis in DCs. We show that SIRT2 is crucial for both clearance of intracellular *Salmonella* and suppression of CD8^+^ T cell response. Overall, the current study suggests, inhibition of SIRT2 during *Salmonella* infection shows a protective role *in vivo*.

## Results

### SIRT2 expression in dendritic cells during *Salmonella* infection

**Fig 1:**
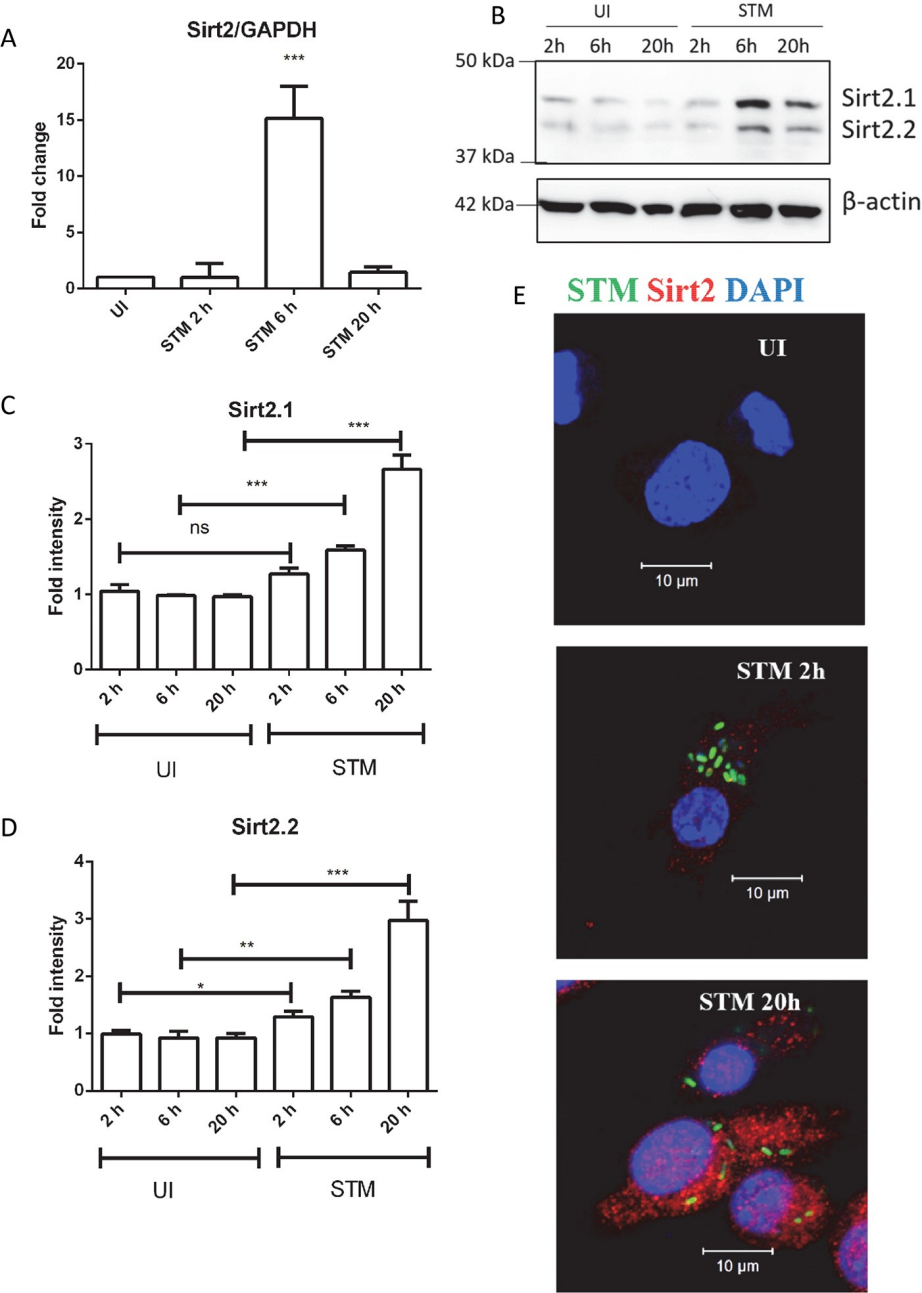
*Salmonella* infection upregulates SIRT2 expression in DCs. A. qPCR analysis of SIRT2 expression in DCs at indicated time post infection. GAPDH was used as an internal control. (UI- Uninfected, STM- *Salmonella* Typhimurium infected) (Data are presented as mean ± SEM of 3 independent experiments). B. Representative immunoblot of SIRT2 in DC at indicated time post- infection. (UI- Uninfected, STM- *Salmonella* Typhimurium infected). C. Densitometry analysis of SIRT2.1 band at indicated time post- infection. (UI- Uninfected, STM- *Salmonella* Typhimurium infected) (Data are presented as mean ± SEM of 3 independent experiments). D. Densitometry analysis of SIRT2.2 band at indicated time post- infection. (UI- Uninfected, STM- *Salmonella* Typhimurium infected) (Data are presented as mean ± SEM of 3 independent experiments). E. Representative immunofluorescence image of DCs showing SIRT2 expression at 20 h post infection (UI- Uninfected, STM- *Salmonella* Typhimurium infected) (Green color- *Salmonella* Typhimurium, Red color- SIRT2, Blue color- DAPI) (unpaired two tailed Student’s t- test, p- value, *** < 0.0001, **<0.001, *<0.01).

In order to understand the effect of *Salmonella* infection on SIRT2 expression, we infected bone marrow-derived dendritic cells (DCs) with *Salmonella*. SIRT2 mRNA is detected in both infected and uninfected DCs. To understand the expression profile of SIRT2 during the course of infection, we have infected DCs with *Salmonella* and monitored its mRNA expression. The qPCR results demonstrate that *Salmonella* infection upregulates SIRT2 mRNA (Fig 1A). Once transcription upregulation of SIRT2 was confirmed, we analyzed the levels of SIRT2 protein by subjecting the whole cell protein lysate to immunoblotting. We have detected two isoforms of SIRT2 at the protein level (Fig 1B). As per our knowledge, this is the first instance where SIRT2 expression has been measured in DCs during infection. The existence of multiple isoforms of SIRT2 is in accordance with a previous study [21]. We have also performed immunofluorescence microscopy to assess SIRT2 expression. *Salmonella* infection results in upregulation of SIRT2 in DCs at 20 h post- infection (Fig 1E). To understand the kinetics of SIRT2 expression during the course of infection, we have measured the mRNA and protein levels at different time intervals. In the transcript level, SIRT2 shows a 15-fold upregulation at 6 h post infection compared to control (Fig 1A). Both isoforms of SIRT2 are upregulated in response to *Salmonella* infection at protein level (Fig 1B, 1C and 1D). The upregulation of SIRT2 expression in due course of infection suggests its involvement in bacterial pathogenesis.

### Role of SIRT2 in intracellular survival of *Salmonella* and in antigen presentation

**Fig 2:**
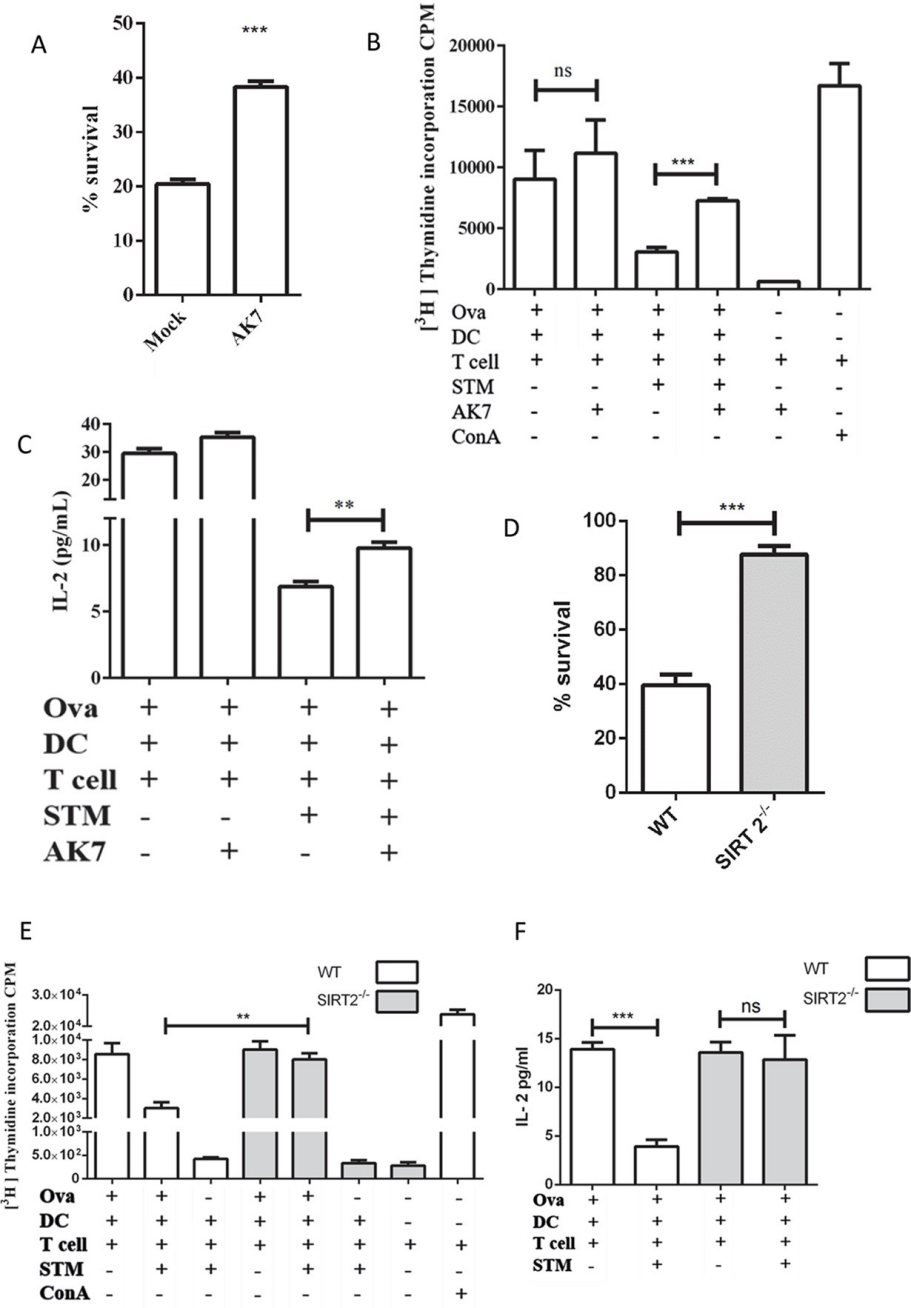
Inhibition of SIRT2 enhances intracellular bacterial survival and CD8^+^T cell response. A. Percentage survival of intracellular *Salmonella* in DCs in gentamicin protection assay in the presence and absence of SIRT2 inhibitor, AK7. (Mock- DMSO treated, AK7- 10 μM AK7 treated). (Data are presented as mean ± SEM of 4 independent experiments) B. ^3^[H] Thymidine incorporation to assess CD8^+^ T cells proliferation during *Salmonella* infection in response to SIRT2 inhibition. (Ova- Ovalbumin, DC- Dendritic cells, T cell- mixed lymphocyte, STM- *Salmonella* Typhimurium, AK7- 10 μM AK7, ConA- Concanavalin A) (Data are presented as mean ± SEM of 3 independent experiments) C. IL-2 levels during CD8^+^T cells proliferation assay during *Salmonella* infection in response to SIRT2 inhibition. (Ova- Ovalbumin, DC- Dendritic cells, T cell- mixed lymphocyte, STM- *Salmonella* Typhimurium) (Data are presented as mean ± SEM of 2 independent experiments) D. Percentage survival of intracellular *Salmonella* in DCs in gentamicin protection assay in wild type and SIRT^-/-^ DCs. (Data are presented as mean ± SEM of 3 independent experiments) E. ^3^[H] Thymidine incorporation to assess CD8^+^ T cells proliferation during *Salmonella* infection in wild type and SIRT2^-/-^ DCs. (Ova- Ovalbumin, DC- Dendritic cells, T cells- mixed lymphocyte, STM- *Salmonella* Typhimurium, ConA- Concanavalin A) (Data are presented as mean ± SEM of 3 independent experiments) F. IL-2 levels during CD8^+^ T cells proliferation assay in response to *Salmonella* infection in wild type and SIRT2^-/-^ DCs. (Ova- Ovalbumin, DC- Dendritic cells, T cells- mixed lymphocyte, STM- *Salmonella* Typhimurium) (Data are presented as mean ± SEM of 2 independent experiments) (unpaired two-tailed Student’s t- test, p- value, *** < 0.0001, **<0.001, *<0.01).

To delineate the role of SIRT2 in bacterial pathogenesis, we have used AK7, a chemical inhibitor of SIRT2. It is a cell permeable competitive inhibitor of SIRT2 with an IC_50_ = 15.5 μM and binds to the NAD^+^ binding site of SIRT2 [22]. To characterize the role of SIRT2 in intracellular survival of *Salmonella*, infected cells were either treated with 10 μM AK7 or left untreated. Survival of the bacteria was quantified by plating the cell lysate at 2 h and 20 h post- infection. In AK7 treated DCs, the bacteria show a 2-fold increased survival as compared to control (Fig 2A). This is further validated by 2-fold enhanced survival of intracellular bacteria in DCs derived from SIRT2^-/-^ mice in comparison with DCs derived from wild type mice (Fig 2D). These results might indicate toward a direct or indirect antimicrobial effect of SIRT2.

During its intracellular life, *Salmonella* resides and proliferates in a modified endosome called *Salmonella*-containing vacuole (SCV). In order to successfully clear this pathogen, it is important to have an efficient CD4^+^ T [23–25] and CD8^+^ T [26,27] cell response. CD4^+^ T cells appear to play a vital role in mitigating *Salmonella* infection as mice compromised in CD4^+^ T cell responses show higher organ burden and succumb to the infection faster. This is mainly attributed to IFNγ secretion by CD4^+^ T cell [28]. However, CD8^+^ T cell also plays a protective role in primary infection [27]. A previous study has demonstrated that *Salmonella* infection impairs CD8^+^ T cell response during chronic infection [29]. However, the role of CD8^+^ T cell response during *Salmonella* infection still remains largely unexplored. To assess the CD8^+^ T cell response, we have used OT1 transgenic mice. The CD8^+^ T cell response was measured by the T cell proliferation assay. The OT1 mice (C57BL/6-Tg (TcraTcrb)1100Mjb/J) have transgenic Tcra-V2 and Tcrb-V5 which can recognize the ovalbumin peptide (257–264 amino acid residues). TCR recognition of cognate peptide-MHC class I (only when presented by H2Kb haplotype) complex results in the proliferation of CD8^+^ T cells [30]. This CD8^+^ T cell proliferation can be quantified by ^3^[H] thymidine incorporation. This method is utilized to measure the antigen cross-presentation potential of antigen presenting cells. As a positive control, we have used a known mitogen concanavalin A, to induce T cell proliferation. In this study, bone marrow-derived dendritic cells (BMDC) were treated with either ovalbumin alone or ovalbumin and *Salmonella* Typhimurium. From our experiments, quantification of ^3^[H] thymidine incorporation indicates that infected BMDCs are compromised in inducing T cell proliferation compared to the uninfected control. However, post- infection AK7 treatment shows the rescue of T cell proliferation implicating the inhibitory role of SIRT2 in antigen presentation (Fig 2B). Furthermore, an enhanced antigen presentation observed in DCs isolated from SIRT2^-/-^ mice compared to wild type mice (Fig 2E). IL-2 is a marker of T cell proliferation [31]. Post- infection AK7 treatment shows increased IL-2 production during T cell proliferation assay (Fig 2C, S1 Fig). Similarly, in comparison with wild type DCs, SIRT2^-/-^ DCs show enhanced IL-2 production (Fig 2F).

These results indicate the role of SIRT2 in two counteractive processes: firstly, its beneficial effect on reducing bacterial intracellular survival and secondly, opposing effect on antigen presentation leading to less clearance of the pathogen.

### SIRT2 actions are nitric oxide mediated

**Fig 3:**
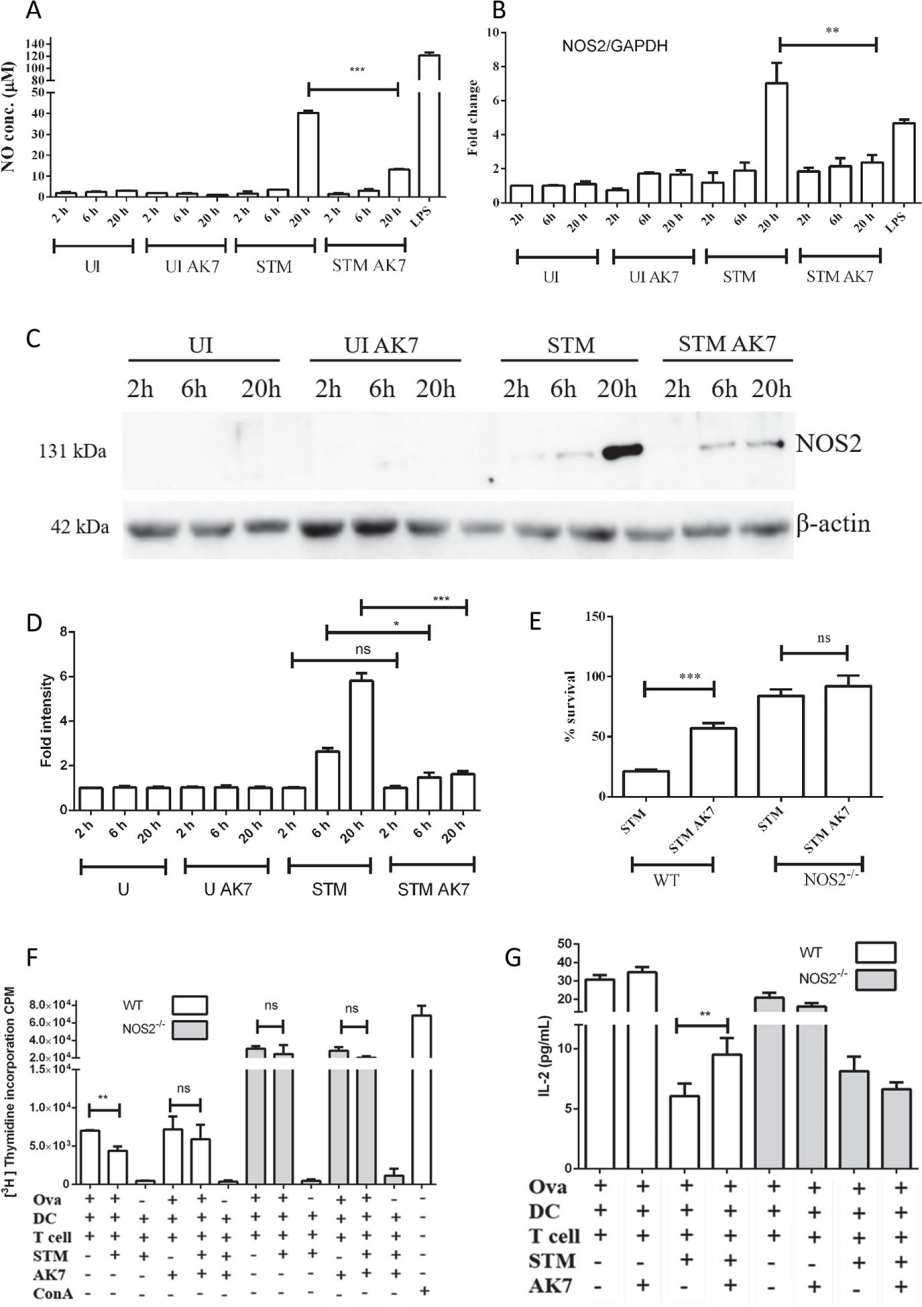
Effect of SIRT2 inhibition is nitric oxide mediated. A. Nitric oxide levels in conditioned media in response to SIRT2 inhibition at indicated time post infection. (UI- uninfected DCs, UI AK7- Uninfected DCs treated with 10 μM AK7, STM- *Salmonella* Typhimurium infected DCs, STM AK7- - *Salmonella* Typhimurium infected DCs treated with 10 μM AK7) (Data are presented as mean ± SEM of 5 independent experiments). B. qPCR analysis of NOS2 expression in DCs at indicated time post infection in response to SIRT2 inhibition. GAPDH was used as an internal control. (UI- uninfected DCs, UI AK7- Uninfected DCs treated with 10 μM AK7, STM- *Salmonella* Typhimurium infected DCs, STM AK7- *Salmonella* Typhimurium infected DCs treated with 10 μM AK7, LPS- 100 ng/ml LPS treated). (Data are presented as mean ± SEM of 3 independent experiments). C. Representative immunoblot of NOS2 in the presence and absence of SIRT2 inhibition at indicated time. (UI- uninfected DCs, UI AK7- Uninfected DCs treated with 10 μM AK7, STM- *Salmonella* Typhimurium infected DCs, STM AK7- *Salmonella* Typhimurium infected DCs treated with 10 μM AK7) D. Densitometry analysis of NOS2 level in the presence and absence of SIRT2 inhibition at indicated time. (UI- uninfected DCs, UI AK7- Uninfected DCs treated with 10 μM AK7, STM- *Salmonella* Typhimurium infected DCs, STM AK7- *Salmonella* Typhimurium infected DCs treated with 10 μM AK7) (Data are presented as mean ± SEM of 3 independent experiments). E. Percentage survival of intracellular *Salmonella* in DCs in gentamicin protection assay in the presence and absence of SIRT2 inhibitor in wild type and NOS2^-/-^ DCs. (Mock- DMSO treated, AK7- 10 μM AK7 treated). (Data are presented as mean ± SEM of 4 independent experiments) F. ^3^[H] Thymidine incorporation assay to assess CD8^+^ T cells proliferation during *Salmonella* infection in wild type and NOS2^-/-^ DCs in the presence and absence of SIRT2 inhibition. (Ova- Ovalbumin, DC- Dendritic cells, T cell- mixed lymphocyte, STM- *Salmonella* Typhimurium, ConA- Concanavalin A) (Data are presented as mean ± SEM of 3 independent experiments) G. IL-2 levels during CD8^+^T cells proliferation assay in response to *Salmonella* infection in wild type and NOS2^-/-^ DCs. (Ova- Ovalbumin, DC- Dendritic cells, T cell- mixed lymphocyte, STM- *Salmonella* Typhimurium) (Data are presented as mean ± SEM of 2 independent experiments) (unpaired two tailed Student’s t- test, p- value, *** < 0.0001, **<0.001, *<0.01).

Previous studies have suggested the role of SIRT2 in inflammation [11,12,32]. SIRT2 has been indicated to play a role in the LPS mediated increase in nitric oxide levels [20]. However, whether SIRT2 positively or negatively regulates nitric oxide production is still controversial. Reports suggest that in microglial cells and macrophages, SIRT2 inhibits nitric oxide production [10,20]. On the contrary, in microglial cells and macrophages, SIRT2 has been reported to enhance NOS2 levels [33,34]. Earlier studies of *Salmonella* pathogenesis have demonstrated definite upregulation of NOS2 expression in response to infection [35,36]. Nitric oxide is a well-known antimicrobial agent [37]. It is one of the innate immune responses against invading pathogen. Further, nitric oxide is also capable of inhibiting T cell proliferation [38]. Taking into account these previous reports as well as results of the current study that showed antimicrobial and immunosuppressive role of SIRT2, next, we decided to test whether these effects are NOS2 mediated. We have measured the nitric oxide levels in the conditioned media using Griess method. AK7 treatment of infected cells resulted in significant reduction in nitric oxide production compared to control (Fig 3A). Further, AK7 treatment of infected cells inhibits upregulation of NOS2 at both the transcriptional level (Fig 3B) and protein level (Fig 3C and 3D). In order to test whether nitric oxide is responsible for antimicrobial effects of SIRT2, we have performed intracellular survival assay in both wild type and NOS2^-/-^ DCs. If the action of SIRT2 is nitric oxide mediated, then AK7 treatment of NOS2^-/-^ DCs should not cause any change in bacterial survival. To test this hypothesis, we have performed intracellular survival assay of *Salmonella* in DCs as indicated previously. Indeed, AK7 treatment of NOS2^-/-^ DC did not show any significant change in percentage bacterial survival as compared to control, whereas, in wild type DCs, AK7 treatment enhances bacterial survival compared to control (Fig 3E). Altogether, these results point to a probable SIRT2 regulated, nitric oxide mediated antimicrobial effect.

Previous literature has suggested the inhibitory role of nitric oxide on T cell proliferation [38]. To test whether the same holds true in this scenario, we have performed T cell proliferation assay in DCs isolated from wild type and NOS2^-/-^ mice as indicated previously. AK7 treatment of infected wild type DCs results in significant enhancement of CD8^+^ T cell proliferation as compared to the control. However, AK7 treatment does not show a change in T cell proliferation in NOS2^-/-^ infected DCs (Fig 3F) and in IL-2 production (Fig 3G). Together, these results hint toward the possibility of SIRT2 regulated immunosuppressive role of nitric oxide.

### SIRT2 is upstream of NOS2

**Fig 4:**
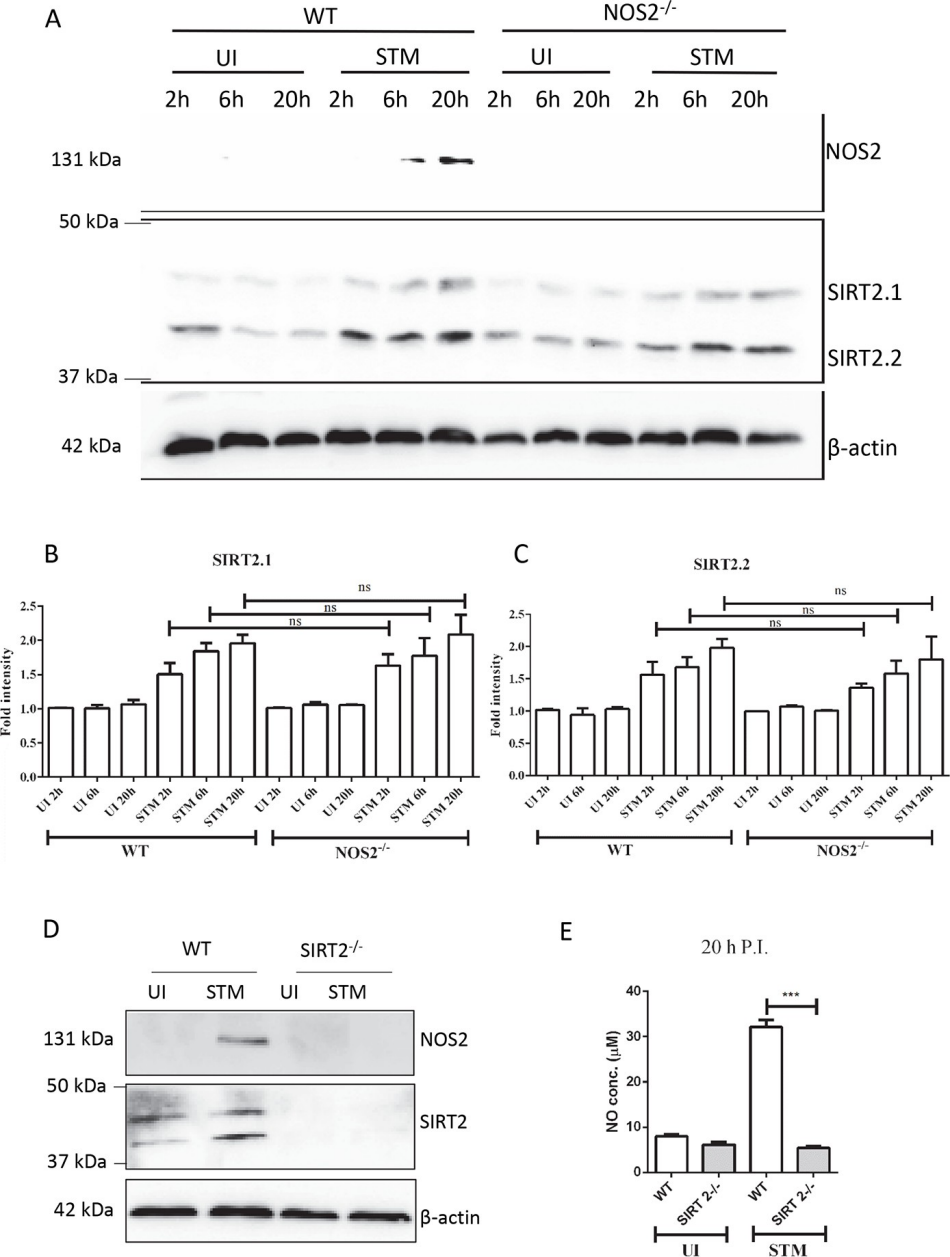
SIRT2 is upstream of NOS2. A. Representative immunoblot of NOS2 and SIRT2 in wild type and NOS2^-/-^ DCs at the indicated time point. (UI- uninfected, STM-*Salmonella* Typhimurium infected) B. Densitometry analysis of SIRT2.1 in wild type and NOS2^-/-^ DCs at the indicated time point. (UI- uninfected, STM-*Salmonella* Typhimurium infected). (Data are presented as mean ± SEM of 2 independent experiments) C. Densitometry analysis of SIRT2.2 in wild type and NOS2^-/-^ DCs at the indicated time point. (UI- uninfected, STM-*Salmonella* Typhimurium infected). (Data are presented as mean ± SEM of 2 independent experiments) D. Representative immunoblot of NOS2 and SIRT2 in wild type and SIRT2^-/-^ DCs at the indicated time point. (UI- uninfected, STM-*Salmonella* Typhimurium infected) E. Nitric oxide levels in conditioned media in response to *Salmonella* infection at 20 h post infection. (UI- uninfected DCs, STM- *Salmonella* Typhimurium infected DCs) (Data are presented as mean ± SEM of 3 independent experiments). (unpaired two-tailed Student’s t-test, p-value, *** < 0.0001, **<0.001, *<0.01).

In the next stage, we tried to decipher whether SIRT2 is upstream or downstream of NOS2. If NOS2 is upstream of SIRT2, then compared to uninfected control, infected NOS2^-/-^ DCs should not show any upregulation of SIRT2, whereas, infected wild type DCs should show SIRT2 upregulation. However, if the reverse holds true, when compared to uninfected control, infected NOS2^-/-^ DCs should upregulate SIRT2 similar to infected wild type DCs. In order to test these two hypotheses, we have infected NOS2^-/-^ DCs and wild type DCs and assessed the SIRT2 levels. Immunoblotting experiments demonstrate that both infected wild type DCs and NOS2^-/-^ DCs upregulate SIRT2 when compared to uninfected control (Fig 4A, 4B and 4C). This confirms that SIRT2 functions upstream of NOS2. This is further supported by the fact that infected SIRT2^-/-^ DCs show no increase in NOS2 level (Fig 4D). Additionally, SIRT2^-/-^ DCs show no significant increase in nitric oxide level in response to *Salmonella* infection (Fig 4E). Further, an initial experiment suggesting that SIRT2 is upstream of NOS2 is shown in Fig 3C, where AK7 inhibits the increase in the level of NOS2 in response to STM infection.

These results confirm that SIRT2 is upstream of NOS2. However, the mechanism involved in SIRT2 mediated regulation of NOS2 needs to be addressed.

### SIRT2 modulates p65 translocation to the nucleus

**Fig 5:**
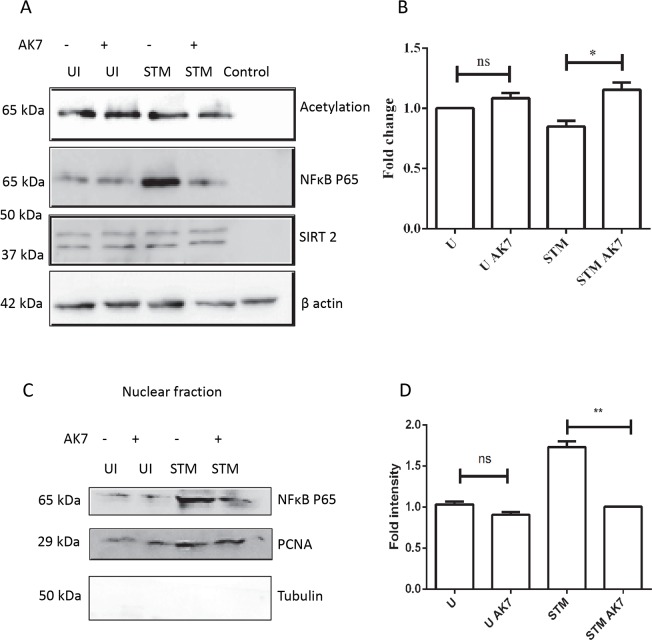
SIRT2 modulates p65 translocation to the nucleus. A. Representative immunoblot of SIRT2 co-IP, probed for NFκB p65 and acetylation in the presence and absence of SIRT2 inhibition at 20 h post infection. (UI- uninfected DCs, UI AK7- Uninfected DCs treated with 10 μM AK7, STM- *Salmonella* Typhimurium infected DCs, STM AK7- *Salmonella* Typhimurium infected DCs treated with 10 μM AK7, Control- Isotype control) (SIRT2^+/+^DCs were used for the purpose of this experiment). B. Densitometry analysis of immunoblot of SIRT2 co-IP, probed for NFκB p65 and acetylation in the presence and in absence of SIRT2 inhibition at 20 h post infection. (UI- uninfected DCs, UI AK7- Uninfected DCs treated with 10 μM AK7, STM- *Salmonella* Typhimurium infected DCs, STM AK7- *Salmonella* Typhimurium infected DCs treated with 10 μM AK7) (Data are presented as mean ± SEM of 3 independent experiments) C. Representative immunoblot of NFκB p65 and PCNA in nuclear isolate. (UI- uninfected DCs, UI AK7- Uninfected DCs treated with 10 μM AK7, STM- *Salmonella* Typhimurium infected DCs, STM AK7- *Salmonella* Typhimurium infected DCs treated with 10 μM AK7) D. Densitometry analysis of immunoblots of NFκB p65 and PCNA in nuclear isolate. (UI- uninfected DCs, UI AK7- Uninfected DCs treated with 10 μM AK7, STM- *Salmonella* Typhimurium infected DCs, STM AK7- *Salmonella* Typhimurium infected DCs treated with 10 μM AK7) (Data are presented as mean ± SEM of 3 independent experiments). (unpaired two-tailed Student’s t-test, p-value, *** < 0.0001, **<0.001, *<0.01).

Apart from its histone deacetylase activity, SIRT2 is capable of deacetylating other protein targets too. These include microtubule [5], FOXO1, 3 [39], NFκB [40] etc. Moreover, various existing reports have demonstrated that NFκB plays a crucial role in regulation of NOS2 [41,42]. A previous study has demonstrated an interaction between p65 and SIRT2 [40]. To test whether SIRT2 interacts with p65, we have performed co-immunoprecipitation of SIRT2 and probed it for p65. Indeed, SIRT2 shows physical interaction with p65 (Fig 5A). Next, we have analysed the acetylation status of co-immunoprecipitated p65. NFκB has four acetylation sites: lysine 218, 221, 310 and 122/123 [43]. Quantification of p65 acetylation indicates that AK7 treatment of infected cells significantly enhances acetylation (Fig 5A and 5B). Depending on the site of acetylation it can either enhance or inhibit nuclear translocation of NFκB p65. Therefore, we have tested for the nuclear translocation of p65 by subcellular fractionation. Infected cells show significantly enhanced nuclear translocation of p65 compared to AK7 treated infected DCs (Fig 5C and 5D). This corroborates with inhibition of NOS2 production in response to AK7 treatment.

**Fig 6:**
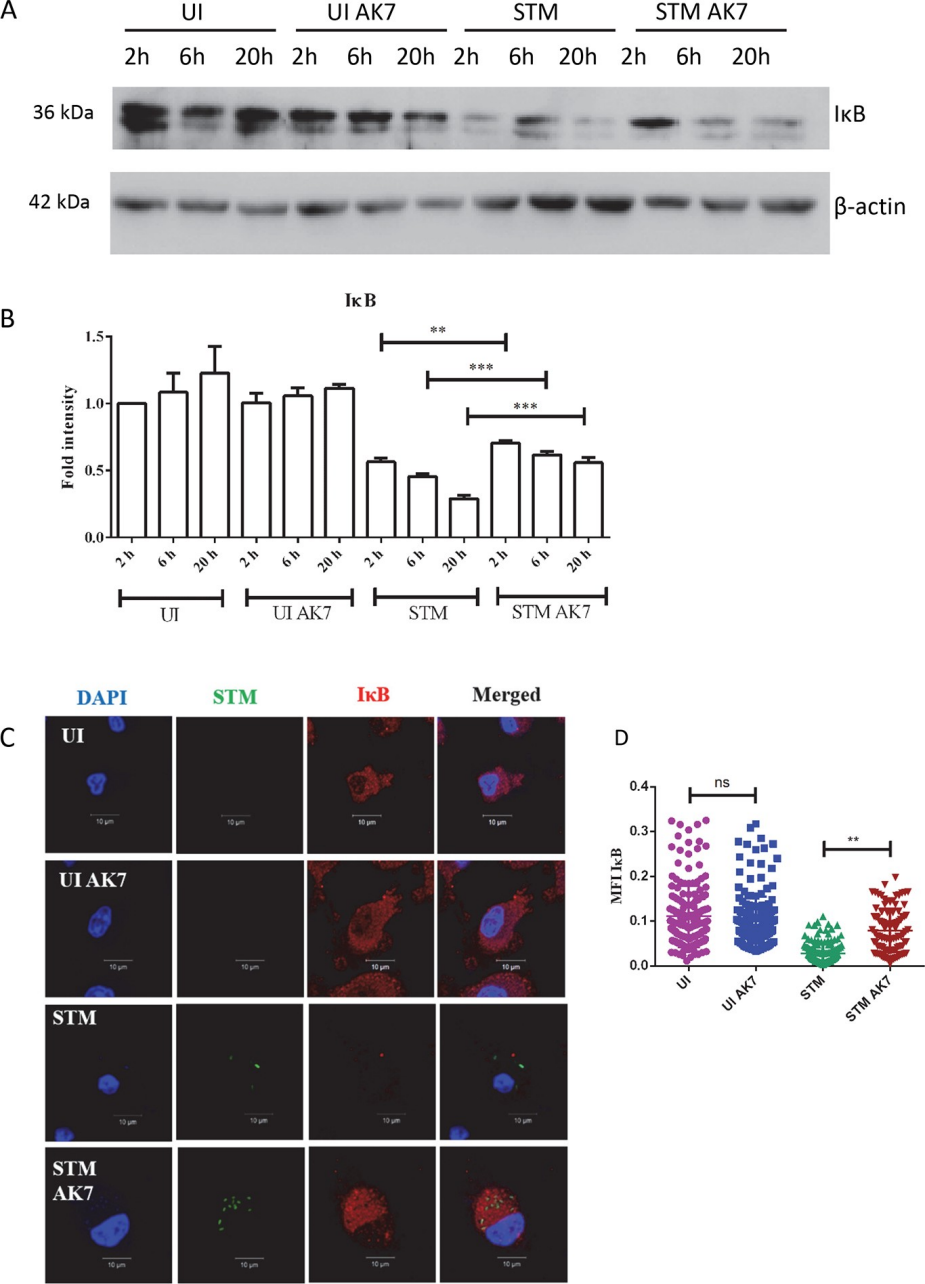
SIRT2 inhibition inhibits IκB degradation. A. Representative immunoblot of IκB in the presence and absence of SIRT2 inhibition at the indicated time point post infection. (UI- uninfected DCs, UI AK7- Uninfected DCs treated with 10 μM AK7, STM- *Salmonella* Typhimurium infected DCs, STM AK7- *Salmonella* Typhimurium infected DCs treated with 10 μM AK7). B. Densitometry analysis of IκB immunoblot in the presence and absence of SIRT2 inhibition at the indicated time point post infection. (UI- uninfected DCs, UI AK7- Uninfected DCs treated with 10 μM AK7, STM- *Salmonella* Typhimurium infected DCs, STM AK7- *Salmonella* Typhimurium infected DCs treated with 10 μM AK7) (Data are presented as mean ± SEM of 3 independent experiments). C. Representative immunofluorescence image of IκB in the presence and absence of SIRT2 inhibition at the indicated time point post infection. (UI- uninfected DCs, UI AK7- Uninfected DCs treated with 10 μM AK7, STM- *Salmonella* Typhimurium infected DCs, STM AK7- *Salmonella* Typhimurium infected DCs treated with 10 μM AK7) D. Mean Fluorescence Intensity (MFI) of immunofluorescence image of IκB in the presence and absence of SIRT2 inhibition at the indicated time point post infection. (UI- uninfected DCs, UI AK7- Uninfected DCs treated with 10 μM AK7, STM- *Salmonella* Typhimurium infected DCs, STM AK7- *Salmonella* Typhimurium infected DCs treated with 10 μM AK7) (Red- IκB, Green- *Salmonella* Typhimurium, Blue-DAPI) (n = 200) (unpaired two-tailed Student’s t- test, p- value, *** < 0.0001, **<0.001, *<0.01).

During its cytosolic localization, NFκB remains bound to IκB. Phosphorylation of IκB with IκB kinase enhances its ubiquitination and hence, proteasomal degradation. This further results in translocation of p65 into the nucleus [44]. Therefore, we have analysed the IκB levels. *Salmonella* infection of DCs induces IκB degradation. Immunoblotting experiments suggest that AK7 treatment of the DCs inhibits this degradation (Fig 6A and 6B). Immunofluorescence study of infected cells also demonstrates AK7 treatment inhibits IκB degradation (Fig 6C and 6D). Together, these results suggest that inhibition of SIRT2 results in inhibition of NFκB translocation thus preventing NOS2 transcription.

### SIRT2 inhibition delays *Salmonella* infection mediated death *in vivo*

**Fig 7:**
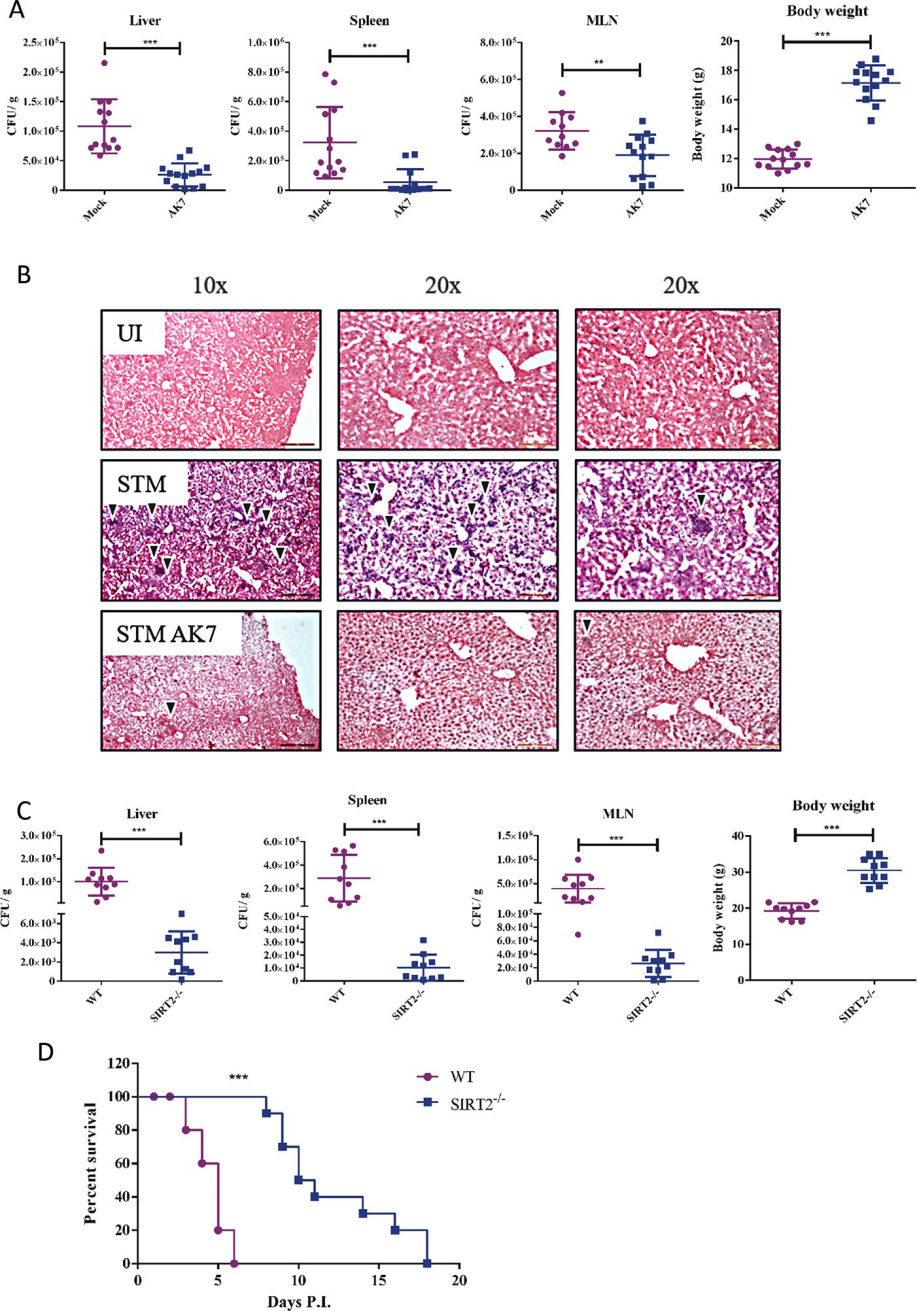
SIRT2 inhibition is protective during *Salmonella* pathogenesis *in-vivo*. A. Organ burden in spleen, liver, MLN and infected mice body weight in presence and absence of SIRT2 inhibitor 5 days post infection. (Mock-only vehicle treated, AK7- 15 mg/kg body weight AK7 was intraperitoneally injected everyday) (Data are presented from 3 independent experiments). B. Representative image of hematoxylin and eosin staining of the liver section to assess *Salmonella* infection mediated liver damage 5 days post infection. (UI- uninfected, STM-*Salmonella* infected, STM AK7- *Salmonella* infected and AK7 treated) C. Organ burden in spleen, liver, MLN and infected mice body weight in wild type and SIRT2^-/-^ mice 5 days post infection. Data are presented from 3 independent experiments. (unpaired two-tailed Student’s t- test, p- value, *** < 0.0001, **<0.001, *<0.01) D. Percentage survival in response to lethal dose of *Salmonella* infection (Log-rank (Mantel-Cox) Test, p- value, *** < 0.0001, **<0.001, *<0.01).

During *Salmonella* infection *ex vivo*, SIRT2 regulates two opposing phenomena namely, intracellular bacterial clearance and suppression of T cell proliferation. However, *in vivo*, whether the innate arm or the adaptive arm of the immune system plays the major role is the question. If nitric oxide mediated clearance of the bacteria is of utmost importance, then, inhibition of SIRT2 should worsen the disease pathology. If CD8^+^ T cell proliferation is of greater importance, in that case, inhibition of SIRT2 should inhibit the disease progression. In order to test this hypothesis C57BL/6 mice were infected with a sub-lethal dose of *Salmonella* Typhimurium and the infected mice were treated everyday with AK7 (15 mg/kg body weight) for 5 days. On the fifth day, mice were sacrificed and the bacterial burden in various organs was quantified by plating. This showed that AK7 treated mice have lesser bacterial burden in the spleen, liver and MLN compared to mock treated mice (Fig 7A, S2 Fig). AK7 treated mice also show higher body weight, compared to control (Fig 7A). Haematoxylin and eosin staining of liver sections showed lesser inflammation in the lobules in the AK7 treated mice compared to untreated mice (Fig 7B). However, we did not observe any change in pro-inflammatory and anti-inflammatory cytokine profile in the serum of wild type AK7 treated and untreated infected mice (S3 Fig). From these results, we can conclude that inhibition of SIRT2 protects mice *in vivo*.

To further confirm the role of SIRT2 in *in vivo* pathogenesis, we have infected both wildtype and SIRT2^-/-^ mice with *Salmonella*. SIRT2^-/-^ mice show lower bacterial burden in spleen, liver and MLN as compared to wildtype mice (Fig 7C, S4A and S4B Fig). However, we did not observe any change in pro-inflammatory and anti-inflammatory cytokine profile in the serum of wild type and SIRT2^-/-^ infected mice (S5 Fig). To evaluate whether SIRT2 deficiency mediated lower systemic bacterial burden, which reflects in the survival of infected mice, we have calculated the percentage survival. In order to do so, we have infected either wild type or SIRT2^-/-^ mice with a lethal dose of *Salmonella*. The percentage survival data demonstrate that SIRT2 ^-/-^ mice survive significantly longer than wild type mice (Fig 7D). Although, SIRT2^-/-^ mice survives longer than wild type mice in response to *Salmonella* infection, eventually, these mice succumb to the infection. It can either be due to an increased bacterial burden or a cytokine storm. To address this question we have accessed both bacterial organ burden and serum cytokine levels at 5 and 10 days post infection. At 10 days post infection, SIRT2^-/-^ mice show reduction in body weight and increase in the bacterial burden in Peyer’s patch but do not show any change in serum cytokine level (S6 Fig, Fig 7). Interestingly, this increased bacterial burden in Peyer’s patch also coincides with the time of death of SIRT2^-/-^ mice in the survival experiment. In totality, these results implicate SIRT2 in antibacterial response during *Salmonella* pathogenesis.

**Fig 8:**
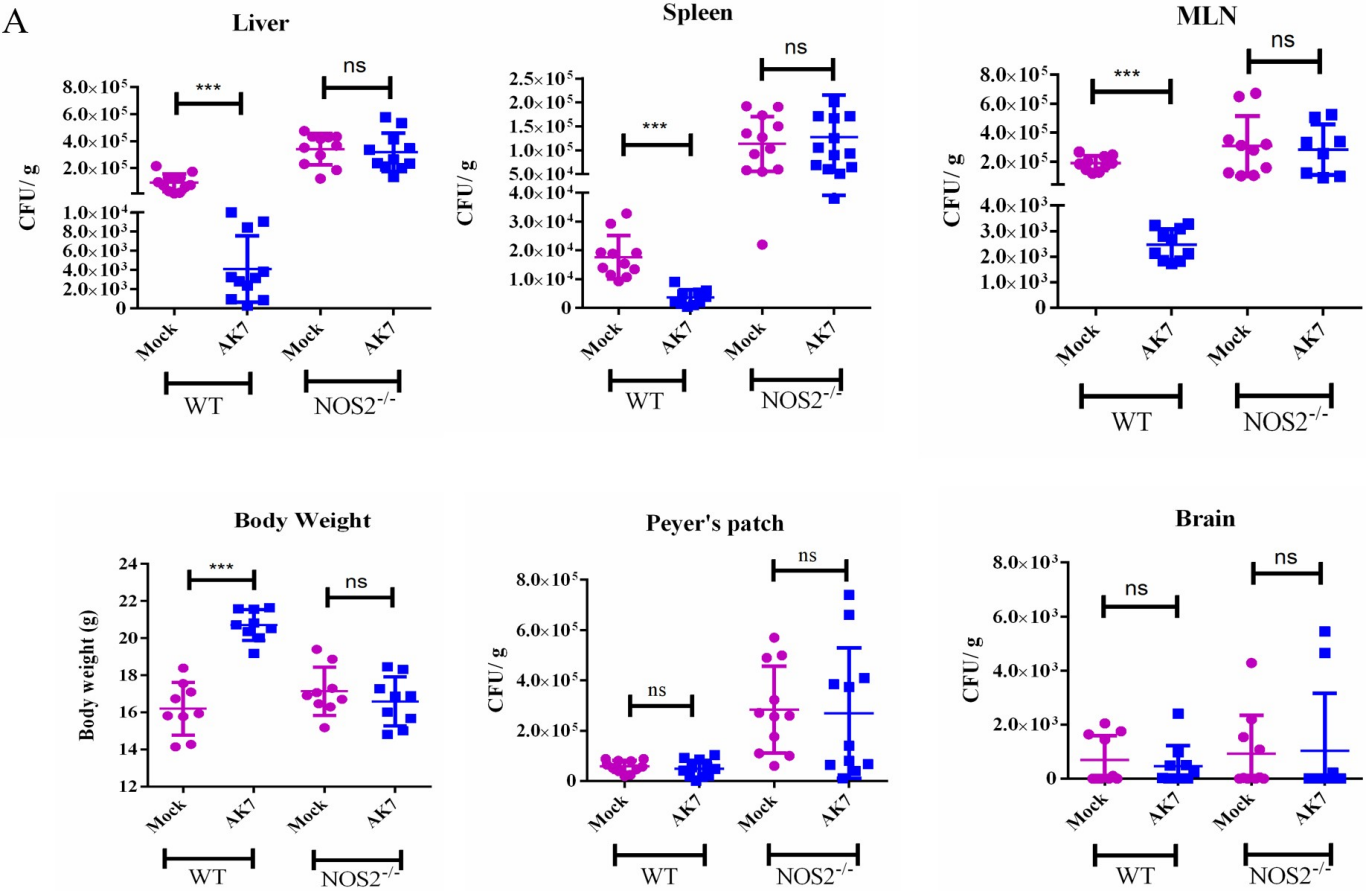
SIRT2 effects *in vivo* is NOS2 mediated. A. Organ burden in WT and NOS2^-/-^ mice liver, spleen, MLN, Peyer’s patch, brain and body weight in infected mice in presence and absence of SIRT2 inhibitor 5 days post infection. (Mock-only vehicle treated, AK7- 15 mg/kg bodyweight AK7 was intraperitoneally injected everyday) (Data are presented from 3 independent experiments).(unpaired two-tailed Student’s t-test, p-value, *** < 0.0001, **<0.001, *<0.01).

*Ex-vivo* experiments have already suggested the role of NOS2 in a SIRT2 mediated manner. To test the hypothesis we have infected NOS2^-/-^ mice with a sub lethal dose of *Salmonella* and either treated them with AK7 or mock. NOS2^-/-^ mice upon AK7 treatment did not show any significant difference in bacterial organ burden as compared to NOS2^-/-^ untreated mice (Fig 8A, S8 Fig). We also did not observe any change in pro-inflammatory and anti-inflammatory cytokine profile in the serum of wild type AK7 treated and untreated infected mice (S9 Fig). This observation indicates towards a probable role of nitric oxide as a SIRT2 mediated effect.

All these results suggest that SIRT2 mediated regulation of immune response is dominant over its effect on bacterial clearance *in vivo*. They also confirm that this effect is NOS2 mediated.

## Discussion

*Salmonella*, a successful pathogen, has co-evolved with its host to circumvent the host immune response. Till now, in terms of sirtuin involvement during *Salmonella* infection, only Ganesan *et al*. have demonstrated the crucial role of SIRT1 in *Salmonella* mediated autophagy [45]. However, the role of other sirtuins during *Salmonella* infection still remains unknown. In this study, we have demonstrated that *Salmonella* infection upregulates SIRT2 expression in DCs. Although the breadth of SIRT2 function remains to be fully characterized, a growing body of literature suggests its role in bacterial infection. Eskandarian *et al*. have recently demonstrated that *Listeria monocytogenes* infection results in translocation of SIRT2 to the nucleus. SIRT2 translocation results in epigenetic modification due to deacetylation of lysine 18 residue of histone 3. It further changes the transcription landscape in the host cells. In case of *Listeria* infection, SIRT2 translocation is mediated by PI3K/AKT signaling pathway in an InlB dependent manner [13]. In the case of mycobacterial infection, SIRT2 ablation results in a transient increase in bacterial burden. However, the long-term outcome does not show any significant difference in myeloid and T cell response [14]. Further, SIRT2 deficient mice show increased survival during *Staphylococcus* infection [46]. These studies provide evidence of a diverse role of SIRT2 in bacterial pathogenesis. To evaluate the role of SIRT2 in the intracellular pathogenesis of *Salmonella* in DCs, we have used bone marrow-derived DCs. In the current study, for the first time, we have shown SIRT2 expression in *Salmonella-*infected DCs. We have observed two isoforms of SIRT2 in DCs. *Salmonella* infection results in upregulation of SIRT2 in DCs. This is the first instance to show that inhibition of SIRT2 leads to better survival of the bacteria in DCs. *Salmonella* has been reported to suppress antigen presentation in DCs but not in other professional antigen presenting cells. Our results suggest that the suppression of CD8^+^ T cell proliferation is SIRT2 mediated. In this current study, both inhibition of bacterial survival and suppression of T cell proliferation is attributed to NOS2 activity. Bueno *et al*. have suggested the link between the intracellular survival of *Salmonella* and antigen presentation by DCs [47]. SIRT2 regulated NOS2 activity might explain the missing link between the intracellular survival of *Salmonella* and T cell proliferation. Our results of *Salmonella* infection mediated upregulation of NOS2 activity in DCs are in accordance with previous reports [35,48]. Further, we also have observed SIRT2 regulated nitric oxide mediated inhibition of CD8^+^ T cell proliferation. This inhibition can either be due to an increase in cell death [49] or due to the induction of T_reg_ cells [50]. Nitric oxide has also been reported to inhibit T_H_17 differentiation [51]. Further study needs to be carried out to address these unanswered questions. Our study does not answer the mechanism by which nitric oxide suppresses T cell proliferation. The possibility of enhanced T cell death is more likely as we do not notice any change in pro-inflammatory and anti-inflammatory cytokine profile in our *in vivo* experiment.

In our study, AK7 treatment enhances the acetylation of NFκB p65. Acetylation plays a crucial role in p65 mediated transcriptional regulation. Acetylation of p65 at 218 and 221 enhances its affinity for DNA binding which results in enhanced expression of p65 response genes. On the contrary, acetylation of lysine 122/123 negatively regulates p65 activity. Therefore, acetylation of p65 can both enhance and inhibit its function. A large set of existing literature has demonstrated p65 mediated transcriptional regulation of NOS2 [52]. In the current study, we have shown AK7 mediated inhibition of NOS2 transcription. Our study showed diminished translocation of p65 to the nucleus in response to AK7 during infection. This might indicate de-repression of p65 due to SIRT2 mediated deacetylation. Further, mass spectrometry analysis of SIRT2 interacting p65 might answer this question. Apart from this, the ratio p65/p50 might also alter in response to AK7 treatment and hence, in the regulation of NOS2 level.

*In vivo* studies have indicated a protective role of SIRT2 inhibitor and protection in SIRT2^-/-^ mice. AK7 has already been tested for its protective role in various neurodegenerative diseases. However, its effect in bacterial infection remains largely unaddressed. Our study demonstrates that SIRT2 deficiency lowers the systemic bacterial burden as well as prolongs survival of infected mice. The effect of SIRT2 on CD4^+^ T cell still remains unaddressed and is beyond the scope of our study. In a recent work by Piracha *et al*. [53], it was found that in actively HBV-replicating cells, SIRT2 upregulation leads to enhanced multiplication of the virus. Higher expression of SIRT2 leads to activation of AKT/GSK3β/β catenin signalling pathway during the infection. It is also known that activation of Wnt signalling increases cytoplasmic β catenin that can condition DCs to produce high levels of IL10, TGFβ and low level of pro-inflammatory cytokines, e,g, IL2, IL6, IL12 etc.[54], which was also observed in our study. These regulatory DCs also induces regulatory T cell response during various autoimmune disease [55,56]. Ciarlo *et al*. have reported the protective role of SIRT2 deficiency during chronic staphylococcal infection. SIRT2^-/-^ mice show prolonged survival and better bacterial clearance as compared to wild type mice in chronic staphylococcal infection [46]. Observations obtained during our current study also show similar results. Ciarlo *et al*. have also suggested that absence of SIRT2 enhances the phagocytosis by the macrophages [46]. While this has suggested a role of autophagy in clearance of the extracellular bacteria *Staphylococcus aureus* in macrophages, mechanism of clearance of intracellular bacteria such as *Salmonella* from DCs still remains unexplored. Considering all the available data, administration of SIRT2 inhibitor or NOS2 inhibitor along with conventional antibiotics might be helpful in clearing persistent infection. As inhibition of SIRT2 leads to better CD8^+^ T cell response it can be also beneficial in treating asymptomatic carriers.

## Materials and methods

### Ethics statement

The animal experiments were carried out in accordance with the approved guidelines of the Institutional Animal Ethics Committee at Indian Institute of Science, Bangalore, India (Registration No: 48/1999/CPCSEA). The Committee for the Purpose of Control and Supervision of Experiments on Animals (CPCSEA) is a statutory Committee, which is established under Chapter 4, Section 15(1) of the Prevention of Cruelty to Animals Act 1960. All procedures involving the use of animals were performed according to Institutional Animal Ethics Committee (IAEC) approved protocol by CPCSEA.

### Cell culture, antibodies and primers

Bone marrow-derived dendritic cells were cultured as previously described [19]. Briefly, bone marrow from the femurs and tibia were collected aseptically from C57BL/6 mice. Cells were cultured for 6 days in RPMI-1640 containing 10% heat-inactivated FBS, 20 ng/ml mGM-CSF (Peprotech), 50 μM β-mercaptoethanol, 100 U/ml penicillin and 100 μg/ml streptomycin. Cells were incubated at 5% CO_2_ and 37°C. After 3 days, mGM-CSF containing media was supplemented. Loosely adherent cells were collected on the sixth day and used for the further experiment [57]. Percentage purity of DCs was 65% to 80%.

Total splenocytes were isolated from the spleen of C57BL/6-Tg (TcraTcrb) 1100Mjb/J mice by mechanical disruption. Erythrocytes were lysed by RBC lysis buffer (Sigma) and cells were maintained in RPMI-1640 containing 10% heat-inactivated FBS. Finally, non-adherent cells were collected and were used for mixed lymphocyte proliferation assay.

Anti SIRT2 antibody (Boster, #PA2283), anti NFκB p65 antibody (Cell Signalling, #3034), anti NOS2 antibody (Bimol, #Cay160862), anti Iκα antibody (Santa Cruz Biotech, #A2208) was used for immunoblot and immunofluorescence analysis.

### Bacterial strains and infection

*Salmonella* Typhimurium 14028 (ATCC) were grown overnight at 37 ^0^C, 160 rpm. DCs were infected with stationary-phase bacterial culture with MOI 10. To synchronize the infection, tissue culture plates were subjected to centrifugation at 500xg for 5 min. Cells were washed with PBS and were treated with RPMI-1640 + 10% FBS containing 50 μg/ml gentamicin for 1 hr. Subsequently, the gentamicin concentration was reduced to 10 μg/ml and maintained until the cells were harvested. AK7 (10 nM) was added along with RPMI-1640 containing 10% FBS, 10 μg/ml gentamicin and maintained until the cells were harvested.

### Immunofluorescence microscopy

Cells were fixed with 3.5% paraformaldehyde for 15 min. All staining was performed in the presence of a permeabilizing agent, 0.5% saponin (Sigma) dissolved in 2.5% BSA containing PBS unless mentioned otherwise. All immunofluorescence images were obtained using Zeiss LSM 750 and were analyzed using ZEN black 2012 platform.

### Co-Immunoprecipitation and immunoblotting

For co-immunoprecipitation, DCs were infected with MOI 10 as mentioned previously. After 10 h post infection, cells were washed with PBS and were lysed in native lysis buffer containing 1% Nonidet P-40, 20 mM Tris (pH 8), 2 mM EDTA, 150 mM NaCl and protease inhibitors mixture (Roche Diagnostics) for 30 min at 4°C. Cell debris was removed by centrifugation at 10,000 rpm for 10 min and the supernatant was treated with anti-SIRT2 antibody (Boster). Antibody-SIRT2 complexes were immunoprecipitated using Protein G linked Sepharose beads (Sigma). Beads were extensively washed with washing buffer and denatured at 95 ^o^C for 10 min. Denatured precipitates were subjected to SDS-PAGE (12% gel) followed by transfer to 0.45 μ PVDF membrane. The membrane was blocked using 5% skimmed milk in TTBS for 1h at room temperature. ECL (BioRad) was used for detection and images were captured using ChemiDoc GE healthcare.

All the densitometry analysis was performed using ImageJ platform.

### Nitric oxide production assay

Nitrite, a stable cell permeable metabolite was measured using Griess assay [58]. 3x10^5^ DCs were seeded in 24 well plates and treated as indicated. Cell conditioned media were collected at defined time intervals. 100 μl aliquots of culture supernatant were transferred to the wells of a new flat-bottom 96-well plate and mixed with 50 μl of 1% sulfanilamide and 50 μl of 0.1% naphthylenediamine sequentially. After a 10 min incubation at room temperature, the nitrite concentration was determined by measuring optical density (OD_595_) of each well using a TECAN microplate reader. Sodium nitrite (Sigma) was used as a standard to determine nitrite concentrations in the cell-free medium.

### Nuclear fractionation

DCs were cultured in 6 well plates and treated as indicated. Posttreatment, cells were scraped in ice-cold PBS and harvested by centrifugation at 1500 rpm for 10 mins. The cell pellets were washed with ice-cold PBS and gently resuspended in ice-cold Buffer A (10 mM HEPES pH7.9, 10 mM KCl, 0.1 mM EDTA, 0.1 mM EGTA, 1 mM DTT and 0.5 mM PMSF). After 15 min incubation on ice, cell membranes were disrupted with .01% NP40. The nuclear pellets were recovered by centrifugation at 13, 000 g for 15 min at 4°C. The supernatant was considered as the cytosolic extract. Nuclear pellets were lysed with ice-cold Buffer C (20 mM HEPES pH7.9, 0.4 M NaCl, 1mM EDTA, 1 mM EGTA, 1 mM DTT and 1 mM PMSF) and nuclear extracts were collected after centrifugation at 13,000 g for 20 min at 4°C.

### *In-vivo* experiment

For all experiments, 6–8 weeks old C57BL/6 or SIRT2 knockout mice (JAX stock #012772—B6.129-Sirt2<tm1.1Fwa>/J, procured from the Jackson Laboratories, USA) were used. For organ burden analysis, 6 weeks old C57BL/6 or SIRT2^-/-^ mice were infected with 10^7^ bacteria orally. Infected mice were intraperitoneally injected daily with either 15 mg/kg body weight AK7 or treated with vehicle only. 5 days post infection, mice were sacrificed and bacterial organ load was estimated by plating the tissue homogenates on SS agar plates. For calculating percentage survival, 6 weeks old C57BL/6 or SIRT2^-/-^ mice were infected with 10^8^ bacteria orally and monitored for fatality. The animal experiments were carried out in accordance with the approved guidelines of the institutional animal ethics committee at the Indian Institute of Science, Bangalore, India (Registration No: 48/1999/CPCSEA). All procedures involving the use of animals were performed according to the Institutional Animal Ethics Committee (IAEC)-approved protocol.

### Histology analysis

6 weeks old C57BL/6 or SIRT2^-/-^ mice were infected with 10^7^ bacteria orally. Infected mice were intraperitoneally injected daily with either 15 mg/kg body weight AK7 or treated with vehicle only. 5 days post infection, mice were sacrificed and livers were collected and fixed using neutral buffered saline. The fixed liver was then dehydrated using a gradually increasing concentration of ethanol and embedded in paraffin. 5μm sections were collected on coated plates. Sections were further rehydrated and then stained with hematoxylin and eosin. Images were collected in a Leica microscope.

### ELISA

Estimation of cytokines in conditioned media was performed according to the manufacturer’s instructions. Briefly, 96-well ELISA plates (BD Bioscience) were coated overnight with capture antibody at 4°C. Next day, plates were washed with 0.1% Tween-20 containing PBS and blocked with 10% FBS for 1 h. Following blocking, wells were washed and incubated with 100 μL of test samples (conditioned media) for 2 h at room temperature. Subsequently, plates were washed and incubated with detection antibody and enzyme reagent for 1 h at room temperature. TMB (Sigma) was used as a substrate and reactions were stopped with 2 N H_2_SO_4_. Absorbance was measured at 450 nm wavelength and the concentration of cytokines was interpolated from a standard curve.

### Quantitative real time PCR

Analysis of gene expression was carried out using qPCR. Briefly, RNA from DCs was isolated using TRIzol (Takara) as per manufacturer’s protocol. The mRNA was reverse transcribed to cDNA using oligo (dT)_18_ primer and Tetro reverse transcriptase (Bioline) as per protocol. The expression profile of target gene was evaluated using specific primers by qPCR master mix (Takara) in Applied Biosystems Vii7 Real time PCR instrument. GAPDH was used as an internal control. For qPCR of SIRT2 forward primer 5’-CACTACTTCATCCGCCTGCT-3’, reverse primer 5’-CCAGCGTGTCTATGTTCTGC-3’, GAPDH forward primer 5’- AGGTCGGTGTGAACGGATTTG-3’, reverse primer 5’- TGTAGACCATGTAGTTGAGGTCA-3’, NOS2 forward primer 5’-CGAAACGCTTCACTTCCAA-3’, reverse primer 5’-TGAGCCTATATTGCTGTGGCT-3’ were used.

### Statistical analysis

Statistical analyses were performed with GraphPad Prism software. Student’s t-test and Log-rank (Mantel-Cox) Test were performed as indicated. The results are expressed as mean ± SD or mean ± SEM. Group sizes, experiment number, and p values for each experiment are described in Figure Legends.

## Supporting information

S1 Fig DCs do not serve as a source of IL-2 during AK7 treatment. IL-2 ELISA result of conditioned media at 2 h and 20 h post- infection (UI- uninfected, UI AK7- uninfected and AK7 treated, STM- *Salmonella* infected, STM AK7- *Salmonella* infected and AK7 treated). (Data are presented as mean ± SEM of 2 independent experiments).
(TIF)

S2 Fig Organ burden in Peyer’s patch and brain in presence and absence of SIRT2 inhibitor 5 days post infection. (Mock-only vehicle treated, AK7- 15 mg/kg bodyweight AK7 was intraperitoneally injected every day) (Data are presented from 3 independent experiments).
(TIF)

S3 Fig SIRT2 inhibition does not change serum cytokine profile in wild type mice. ELISA results of serum TNF- α, IL-2, IL-6 (pro-inflammatory) and IL-4, IL-10 (anti-inflammatory) cytokine profile. (UI- uninfected, UI AK7- uninfected and AK7 treated, STM- *Salmonella* infected, STM AK7- *Salmonella* infected and AK7 treated). (Data are presented as mean ± SD of 3 independent experiments).
(TIF)

S4 Fig A. Organ burden in Peyer’s patch and brain in wild type and SIRT2^-/-^ mice 5 days post infection.
B. Immunoblot of SIRT2 for genotype confirmation.
(TIF)

S5 Fig SIRT2 deletion does not change serum cytokine profile in SIRT2^-/-^ type mice. ELISA results of serum TNF-α, IL-2, IL-6 (pro-inflammatory) and IL-4, IL-10 (anti-inflammatory) cytokine profile. (UI- uninfected, STM- *Salmonella* infected). (Data are presented as mean ± SD of 3 independent experiments).
(TIF)

S6 Fig Organ burden in spleen, liver, MLN, Peyer’s patch, brain and body weight in SIRT2^-/-^ mice on 5 days and 10 days post infection. (TIF)

S7 Fig SIRT2 deletion does not change serum cytokine profile in SIRT2^-/-^ type mice on 5 days and 10 days post infection. ELISA results of serum TNF-α, IL-2, IL-6 (pro-inflammatory) and IL-4, IL-10 (anti-inflammatory) cytokine profile. (UI- uninfected, STM- *Salmonella* infected).
(TIF)

S8 Fig Organ burden in NOS2^-/-^ mice Peyer’s patch and brain in the presence and absence of SIRT2 inhibitor 5 days post infection. (Mock-only vehicle treated, AK7- 15 mg/kg bodyweight AK7 was intraperitoneally injected everyday) (Data are presented from 3 independent experiments).
(TIF)

S9 Fig SIRT2 inhibition does not change serum cytokine profile in NOS2^-/-^ type mice. ELISA results of serum TNF-α, IL-2, IL-6 (pro-inflammatory) and IL-4, IL-10 (anti-inflammatory) cytokine profile. (UI- uninfected, UI AK7- uninfected and AK7 treated, STM- *Salmonella* infected, STM AK7- *Salmonella* infected and AK7 treated). (Data are presented as mean ± SD of 3 independent experiments).
(TIF)
