# Correction: *Salmonella* escapes adaptive immune response via SIRT2 mediated modulation of innate immune response in dendritic cells

**DOI:** 10.1371/journal.ppat.1008345

**Published:** 2020-02-05

**Authors:** Mayuri Gogoi, Kasturi Chandra, Mohsen Sarikhani, Ramya Ramani, Nagalingam Ravi Sundaresan, Dipshikha Chakravortty

There are errors in Fig 3. The β actin loading control blot in Fig 3C was in the wrong orientation in the submission. Please see the correct Fig 3 here.

**Fig 3:**
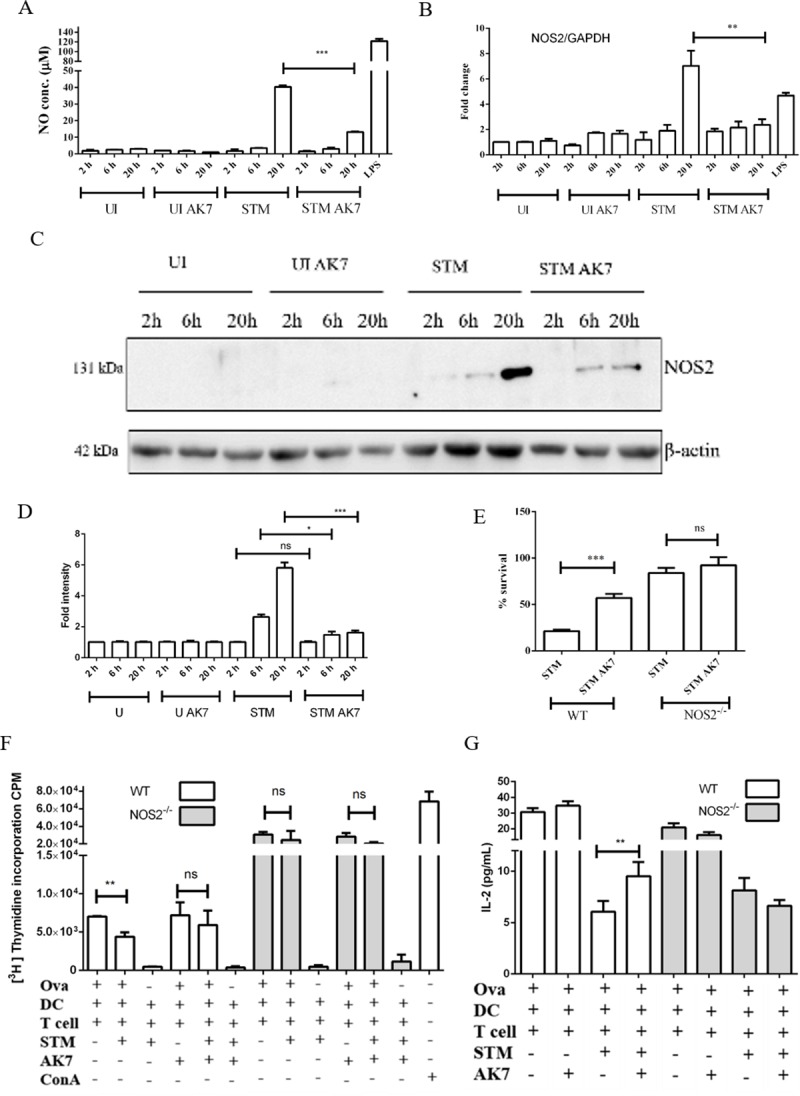
Effect of SIRT2 inhibition is nitric oxide mediated. A. Nitric oxide levels in conditioned media in response to SIRT2 inhibition at indicated time post infection. (UI- uninfected DCs, UI AK7- Uninfected DCs treated with 10 μM AK7, STM- *Salmonella* Typhimurium infected DCs, STM AK7- - *Salmonella* Typhimurium infected DCs treated with 10 μM AK7) (Data are presented as mean ± SEM of 5 independent experiments). B. qPCR analysis of NOS2 expression in DCs at indicated time post infection in response to SIRT2 inhibition. GAPDH was used as an internal control. (UI- uninfected DCs, UI AK7- Uninfected DCs treated with 10 μM AK7, STM- *Salmonella* Typhimurium infected DCs, STM AK7- *Salmonella* Typhimurium infected DCs treated with 10 μM AK7, LPS- 100 ng/ml LPS treated). (Data are presented as mean ± SEM of 3 independent experiments). C. Representative immunoblot of NOS2 in the presence and absence of SIRT2 inhibition at indicated time. (UI- uninfected DCs, UI AK7- Uninfected DCs treated with 10 μM AK7, STM- *Salmonella* Typhimurium infected DCs, STM AK7- *Salmonella* Typhimurium infected DCs treated with 10 μM AK7) D. Densitometry analysis of NOS2 level in the presence and absence of SIRT2 inhibition at indicated time. (UI- uninfected DCs, UI AK7- Uninfected DCs treated with 10 μM AK7, STM- *Salmonella* Typhimurium infected DCs, STM AK7- *Salmonella* Typhimurium infected DCs treated with 10 μM AK7) (Data are presented as mean ± SEM of 3 independent experiments). E. Percentage survival of intracellular *Salmonella* in DCs in gentamicin protection assay in the presence and absence of SIRT2 inhibitor in wild type and NOS2^-/-^ DCs. (Mock- DMSO treated, AK7- 10 μM AK7 treated). (Data are presented as mean ± SEM of 4 independent experiments) F. ^3^[H] Thymidine incorporation assay to assess CD8^+^ T cells proliferation during *Salmonella* infection in wild type and NOS2^-/-^ DCs in the presence and absence of SIRT2 inhibition. (Ova- Ovalbumin, DC- Dendritic cells, T cell- mixed lymphocyte, STM- *Salmonella* Typhimurium, ConA- Concanavalin A) (Data are presented as mean ± SEM of 3 independent experiments) G. IL-2 levels during CD8^+^T cells proliferation assay in response to *Salmonella* infection in wild type and NOS2^-/-^ DCs. (Ova- Ovalbumin, DC- Dendritic cells, T cell- mixed lymphocyte, STM- *Salmonella* Typhimurium) (Data are presented as mean ± SEM of 2 independent experiments) (unpaired two tailed Student’s t- test, p- value, *** < 0.0001, **<0.001, *<0.01).
