# Supplementary figures and images for: Salmonella escapes adaptive immune response via SIRT2 mediated modulation of innate immune response in dendritic cells
Source: PLoS Pathog. 2018 Nov 19;14(11):e1007437. doi: 10.1371/journal.ppat.1007437 (PMC6277114; doi:10.1371/journal.ppat.1007437)

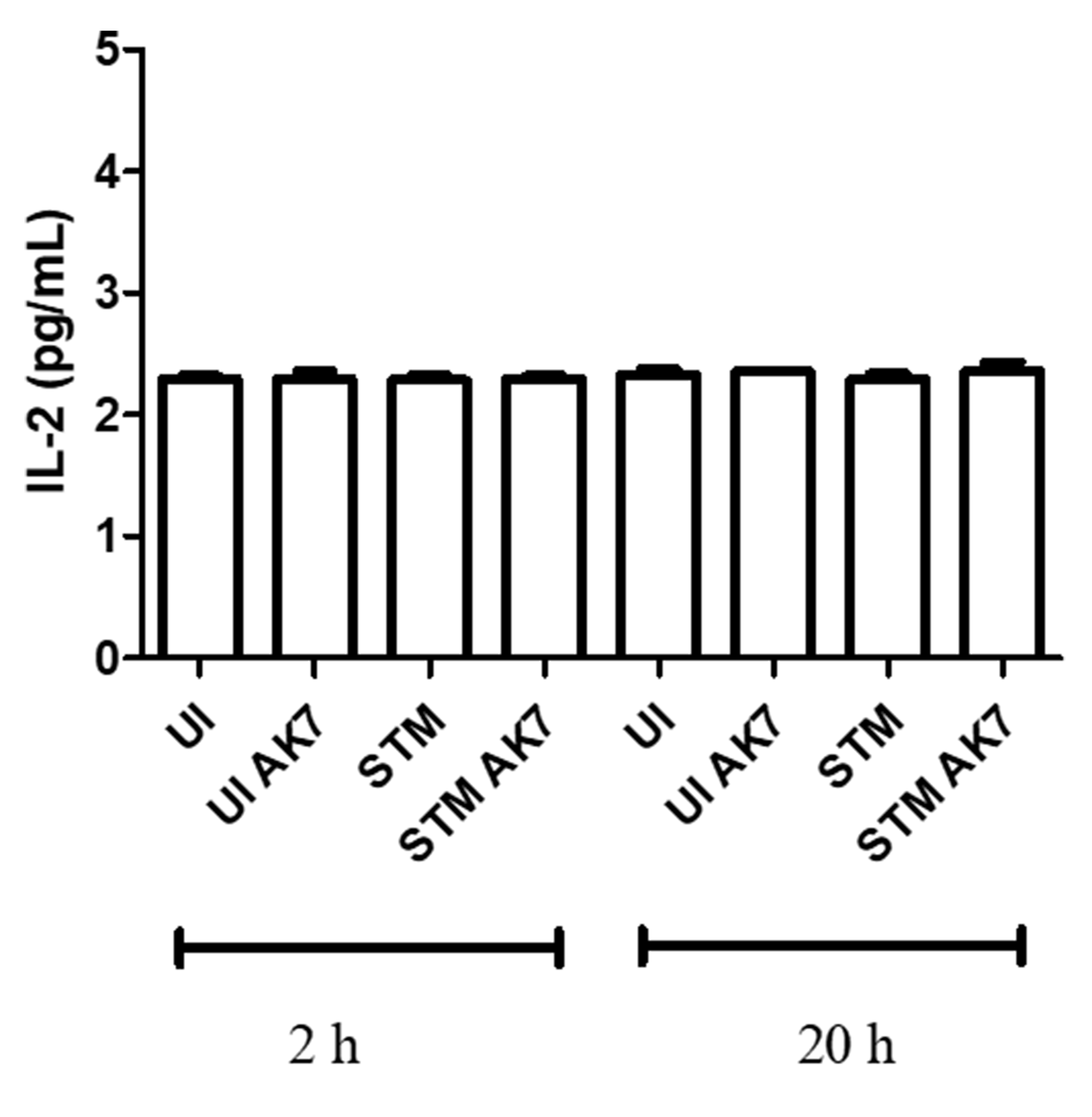

Supplement: S1 Fig — IL-2 ELISA result of conditioned media at 2 h and 20 h post- infection (UI- uninfected, UI AK7- uninfected and AK7 treated, STM- Salmonella infected, STM AK7- Salmonella infected and AK7 treated). (Data are presented as mean ± SEM of 2 independent experiments). (TIF) [file ppat.1007437.s001.tif]

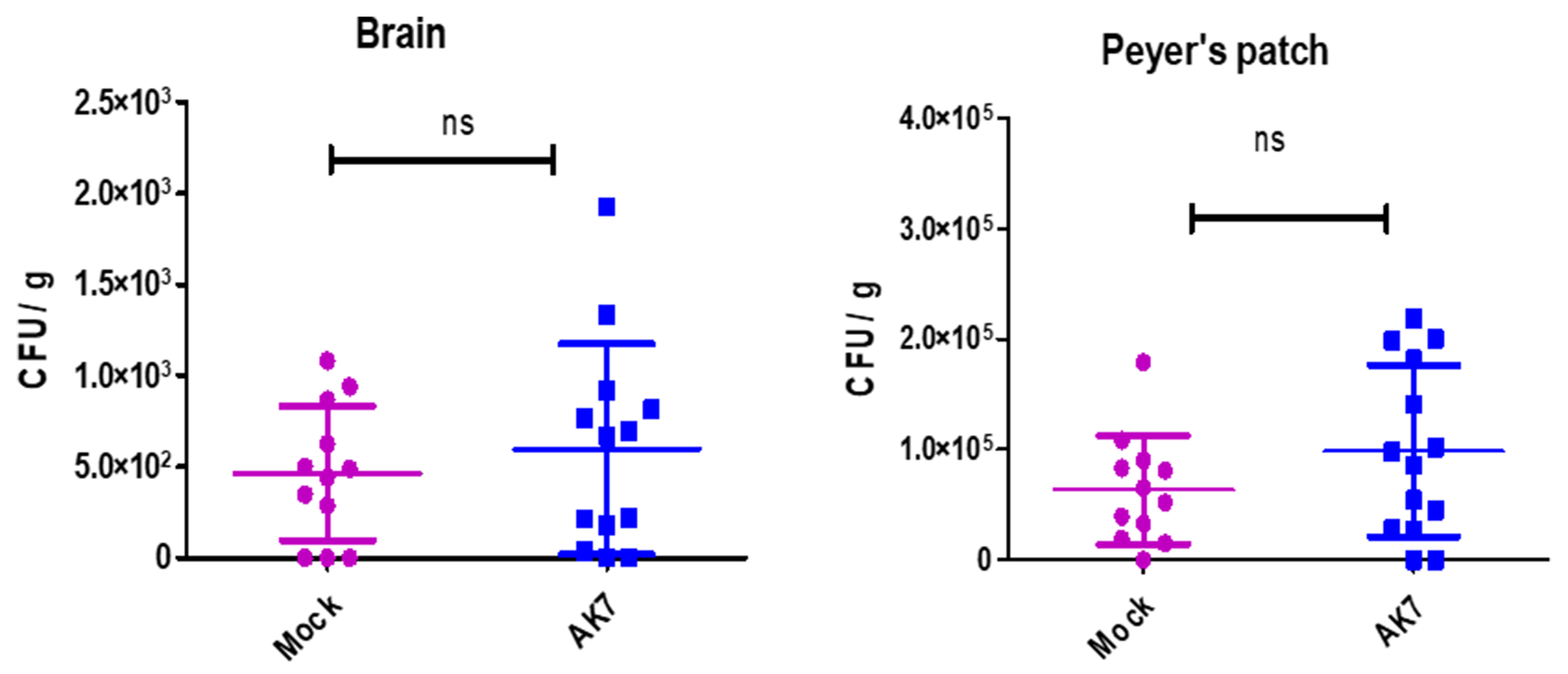

Supplement: S2 Fig — (Mock-only vehicle treated, AK7- 15 mg/kg bodyweight AK7 was intraperitoneally injected every day) (Data are presented from 3 independent experiments). (TIF) [file ppat.1007437.s002.tif]

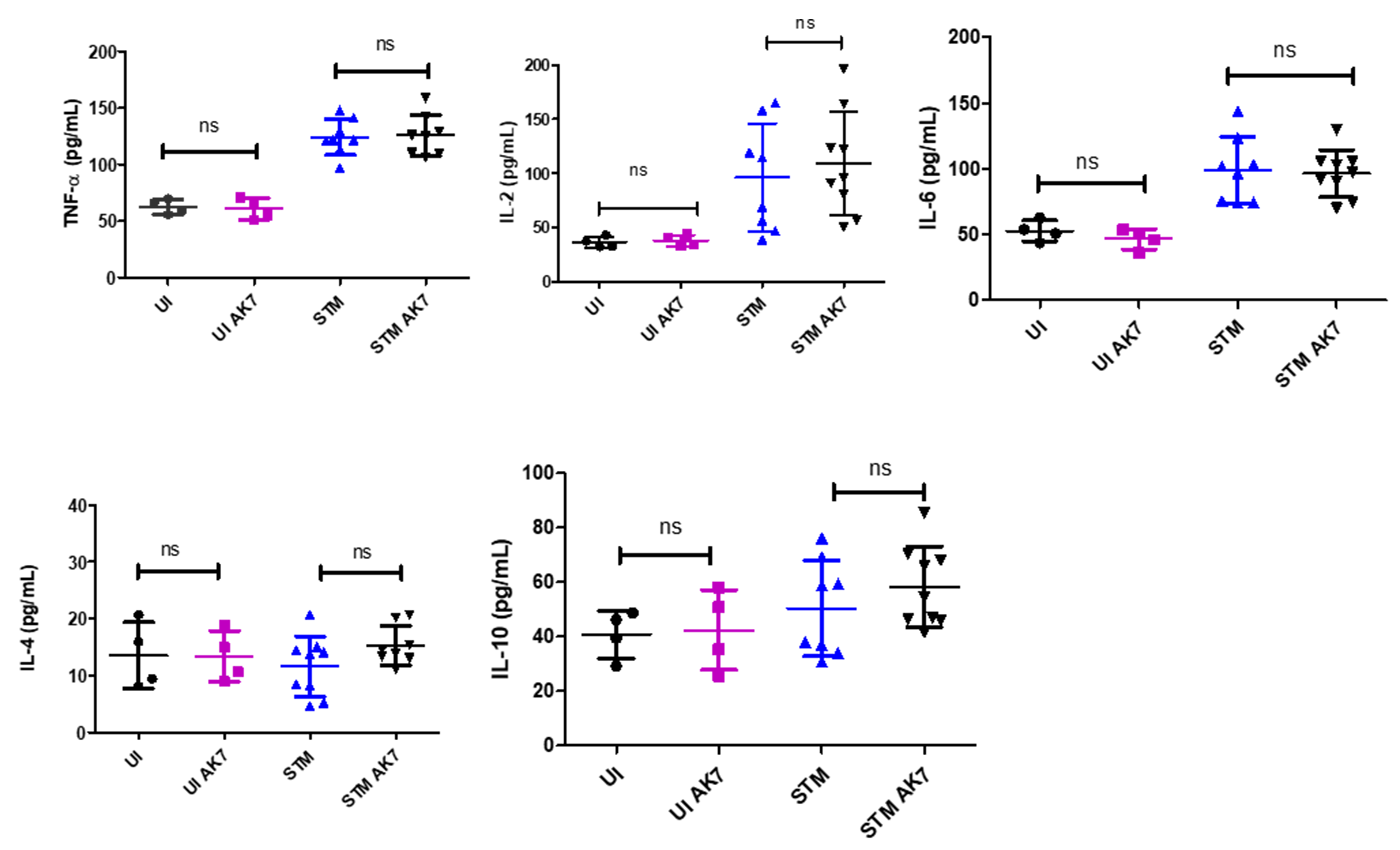

Supplement: S3 Fig — ELISA results of serum TNF- α, IL-2, IL-6 (pro-inflammatory) and IL-4, IL-10 (anti-inflammatory) cytokine profile. (UI- uninfected, UI AK7- uninfected and AK7 treated, STM- Salmonella infected, STM AK7- Salmonella infected and AK7 treated). (Data are presented as mean ± SD of 3 independent experiments). (TIF) [file ppat.1007437.s003.tif]

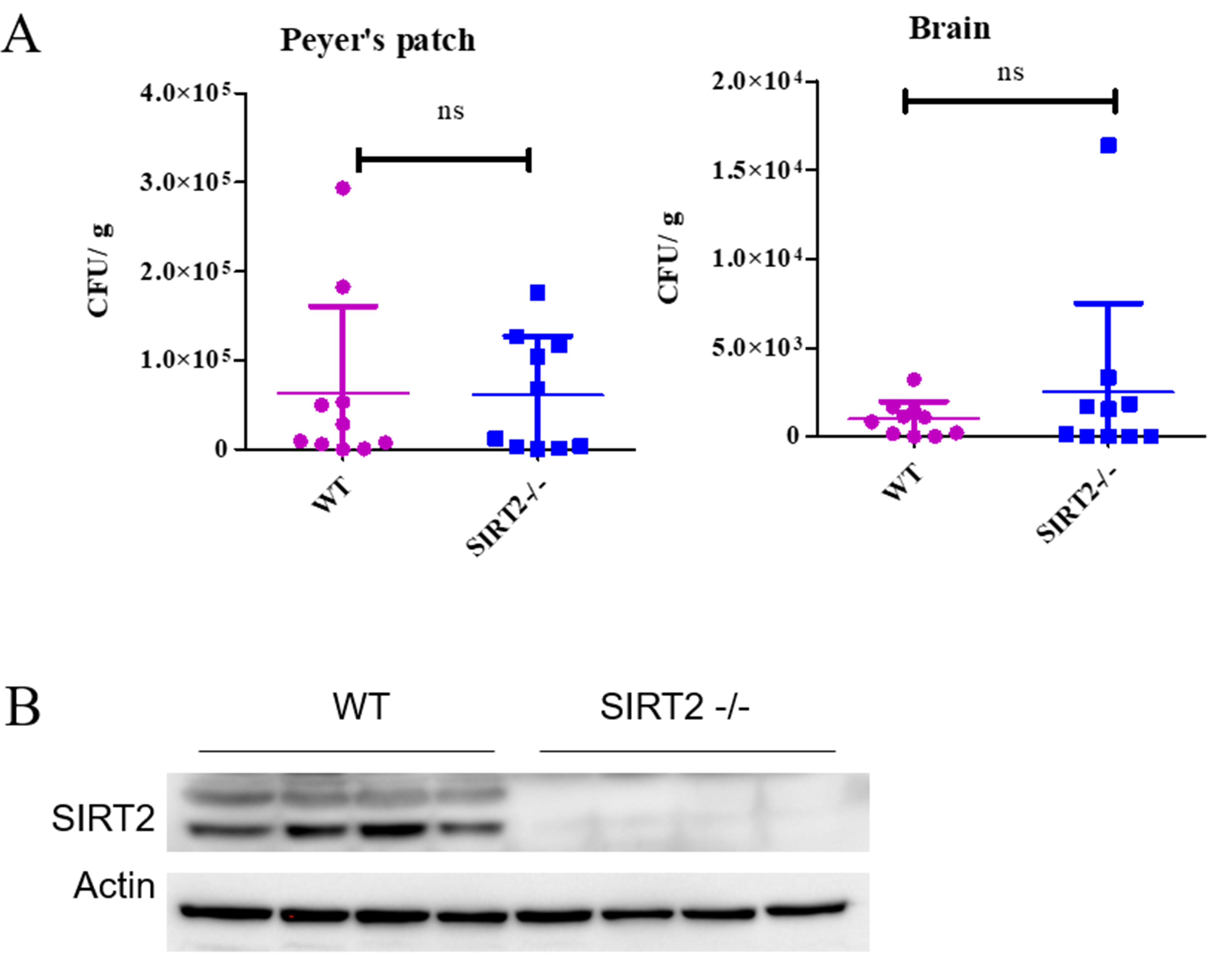

Supplement: S4 Fig — A. Organ burden in Peyer’s patch and brain in wild type and SIRT2-/- mice 5 days post infection. B. Immunoblot of SIRT2 for genotype confirmation. (TIF) [file ppat.1007437.s004.tif]

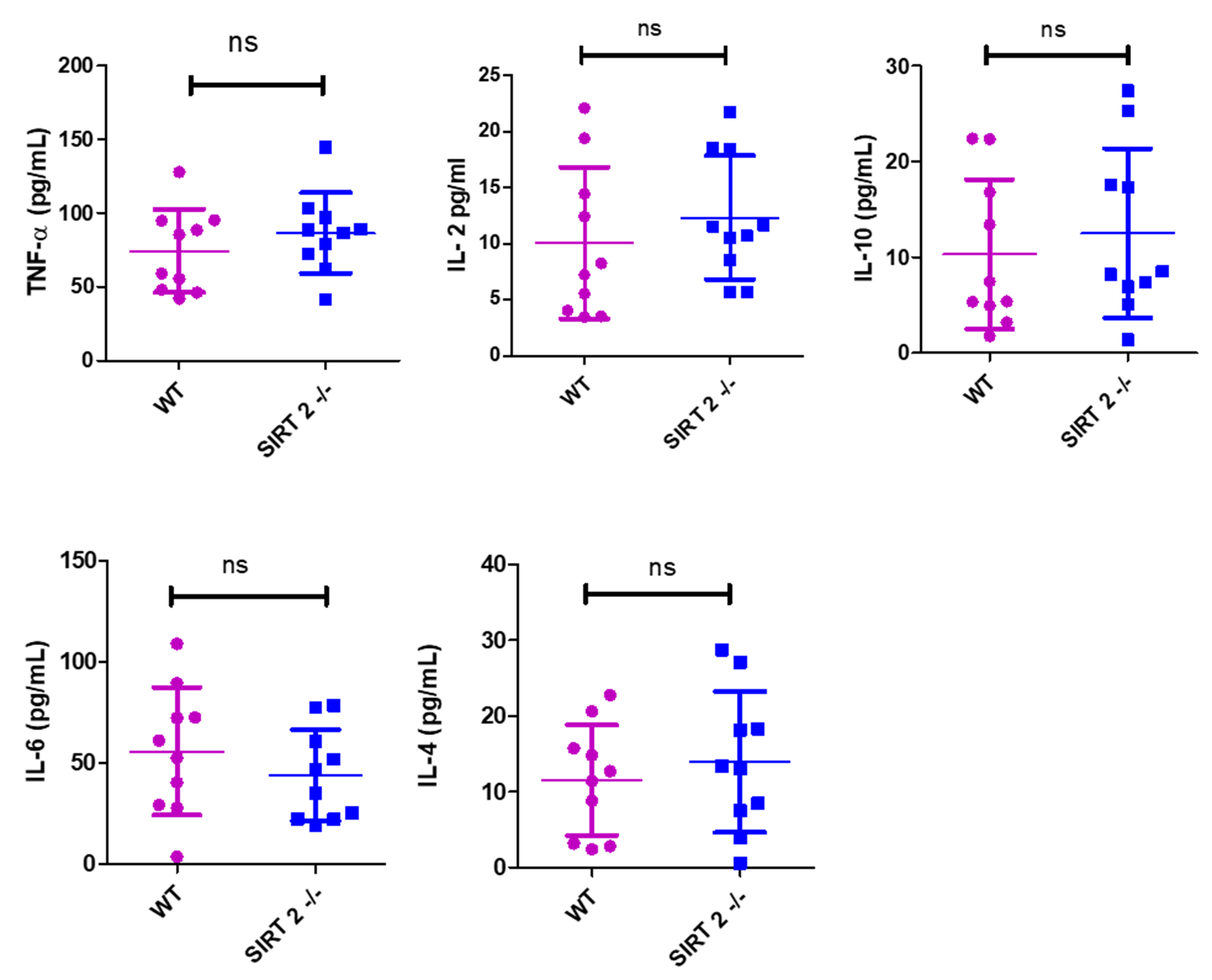

Supplement: S5 Fig — ELISA results of serum TNF-α, IL-2, IL-6 (pro-inflammatory) and IL-4, IL-10 (anti-inflammatory) cytokine profile. (UI- uninfected, STM- Salmonella infected). (Data are presented as mean ± SD of 3 independent experiments). (TIF) [file ppat.1007437.s005.tif]

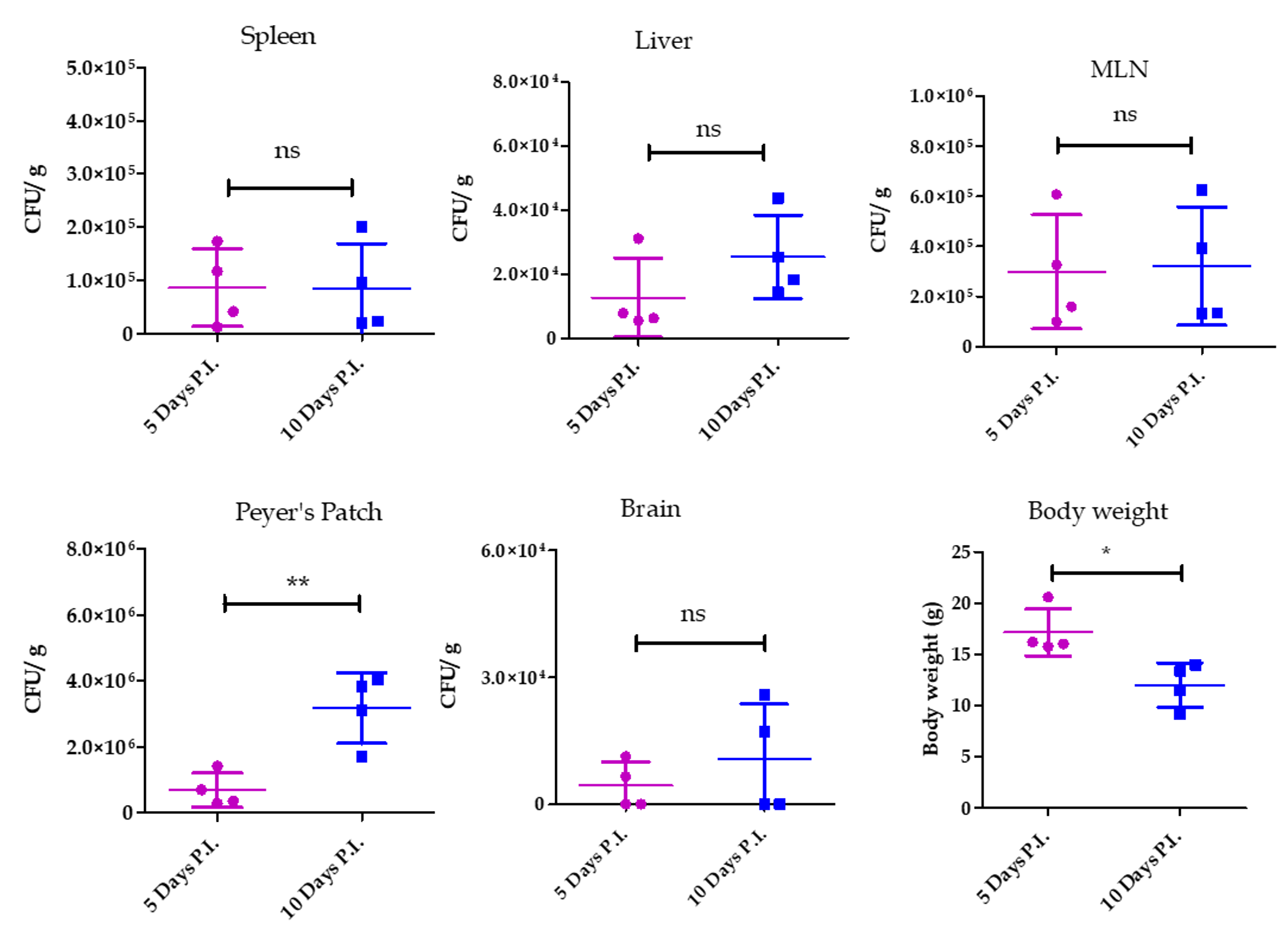

Supplement: S6 Fig — (TIF) [file ppat.1007437.s006.tif]

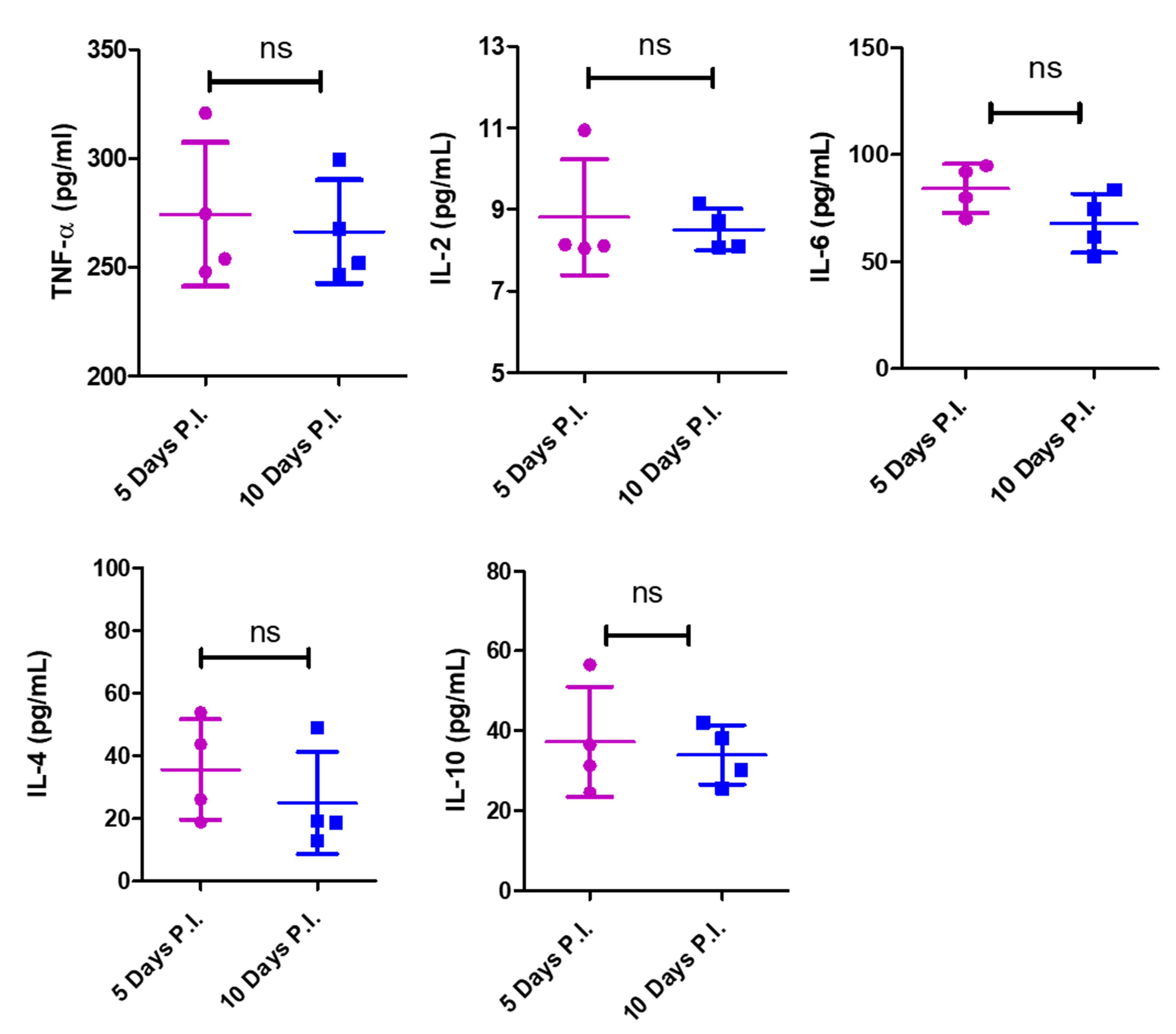

Supplement: S7 Fig — ELISA results of serum TNF-α, IL-2, IL-6 (pro-inflammatory) and IL-4, IL-10 (anti-inflammatory) cytokine profile. (UI- uninfected, STM- Salmonella infected). (TIF) [file ppat.1007437.s007.tif]

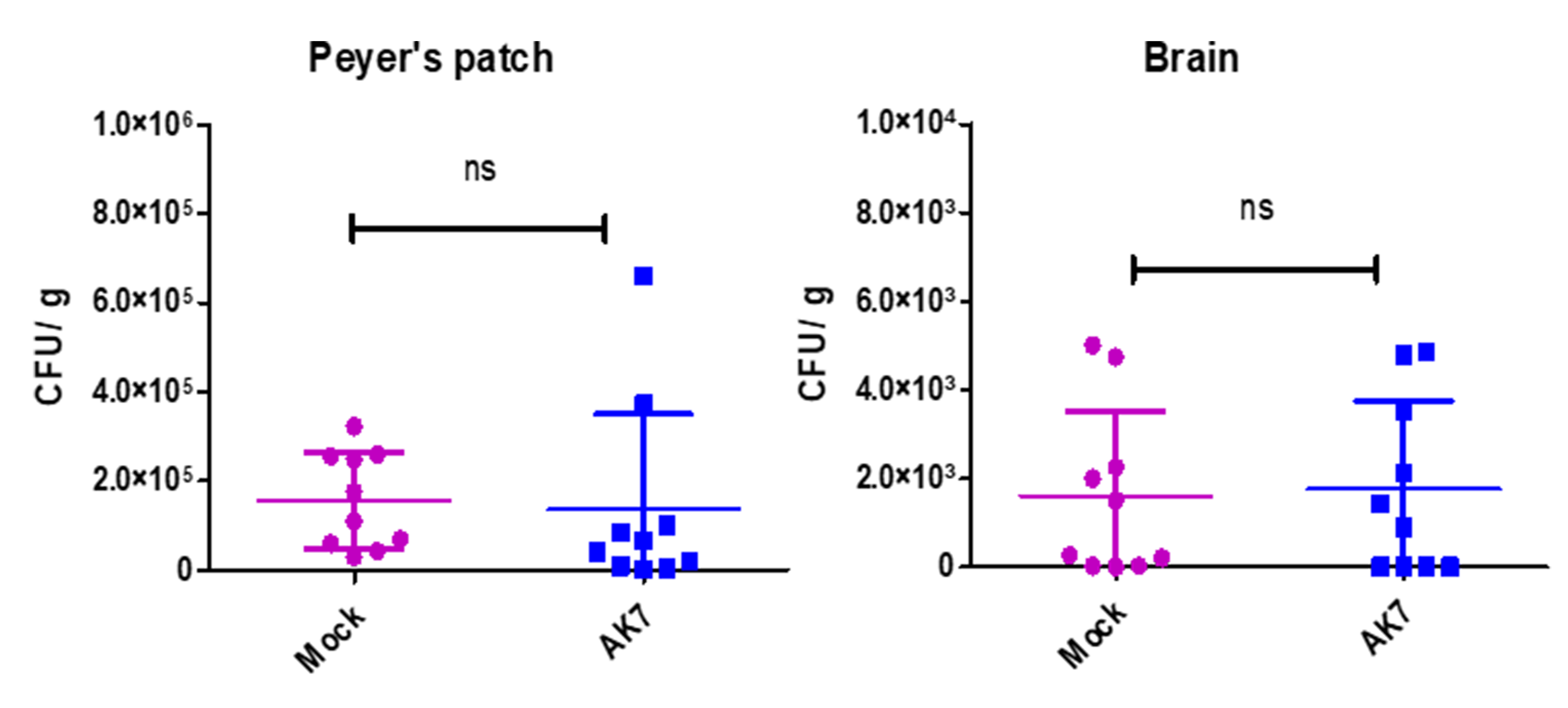

Supplement: S8 Fig — (Mock-only vehicle treated, AK7- 15 mg/kg bodyweight AK7 was intraperitoneally injected everyday) (Data are presented from 3 independent experiments). (TIF) [file ppat.1007437.s008.tif]

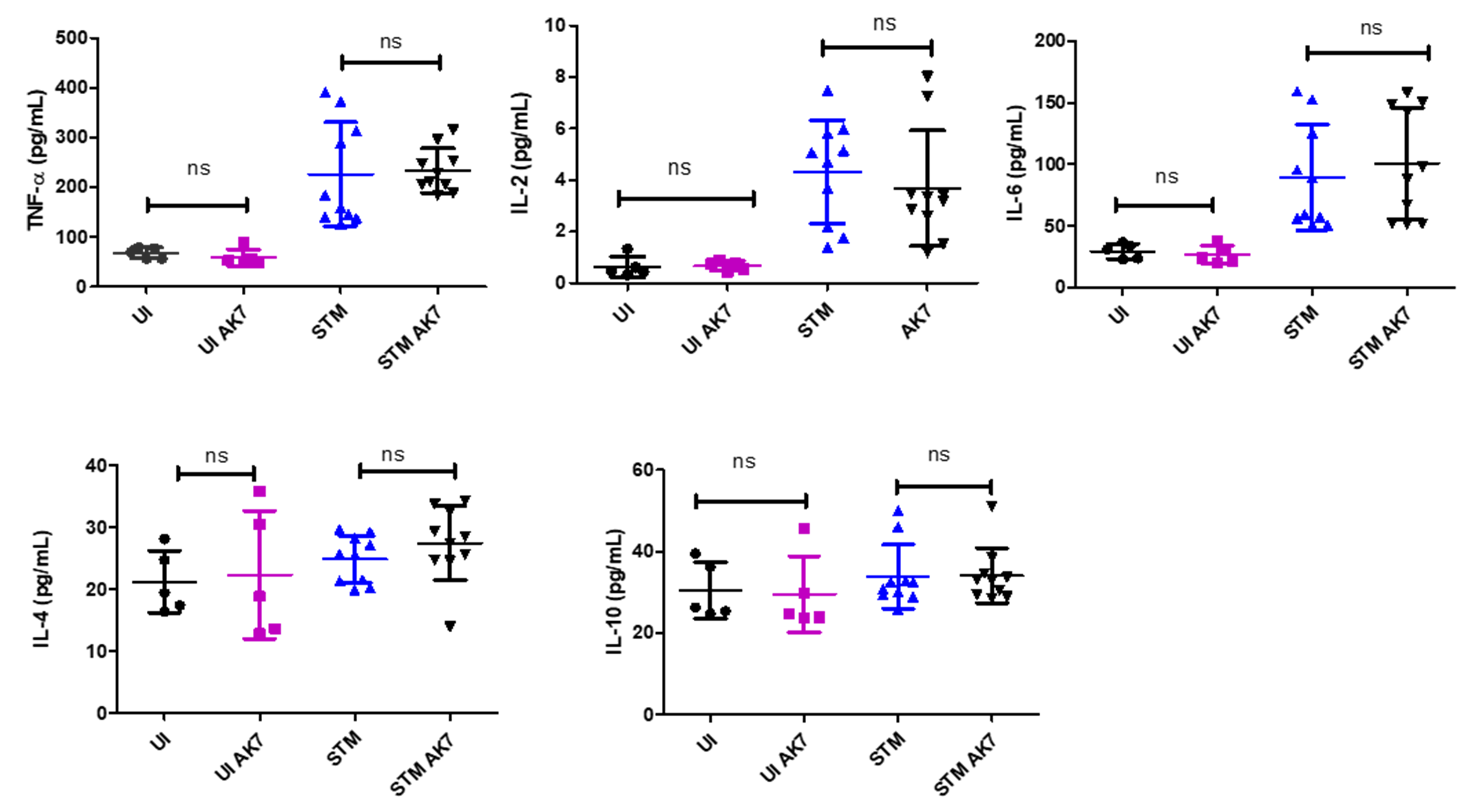

Supplement: S9 Fig — ELISA results of serum TNF-α, IL-2, IL-6 (pro-inflammatory) and IL-4, IL-10 (anti-inflammatory) cytokine profile. (UI- uninfected, UI AK7- uninfected and AK7 treated, STM- Salmonella infected, STM AK7- Salmonella infected and AK7 treated). (Data are presented as mean ± SD of 3 independent experiments). (TIF) [file ppat.1007437.s009.tif]
